# The STIM1-binding site nexus remotely controls Orai1 channel gating

**DOI:** 10.1038/ncomms13725

**Published:** 2016-12-08

**Authors:** Yandong Zhou, Xiangyu Cai, Natalia A. Loktionova, Xianming Wang, Robert M. Nwokonko, Xizhuo Wang, Youjun Wang, Brad S. Rothberg, Mohamed Trebak, Donald L. Gill

**Affiliations:** 1 Department of Cellular and Molecular Physiology, The Pennsylvania State University College of Medicine, Hershey, Pennsylvania 17033, USA; 2 Beijing Key Laboratory of Gene Resources and Molecular Development College of Life Sciences, Beijing Normal University, Beijing 100875, China; 3 Department of Biochemistry, Temple University School of Medicine, Philadelphia, Pennsylvania 19140, USA

## Abstract

The ubiquitously expressed Orai Ca^2+^ channels are gated through a unique process of intermembrane coupling with the Ca^2+^-sensing STIM proteins. Despite the significance of Orai1-mediated Ca^2+^ signals, how gating of Orai1 is triggered by STIM1 remains unknown. A widely held gating model invokes STIM1 binding directly to Orai1 pore-forming helix. Here we report that an Orai1 C-terminal STIM1-binding site, situated far from the N-terminal pore helix, alone provides the trigger that is necessary and sufficient for channel gating. We identify a critical ‘nexus' within Orai1 connecting the peripheral C-terminal STIM1-binding site to the Orai1 core helices. Mutation of the nexus transforms Orai1 into a persistently open state exactly mimicking the action of STIM1. We suggest that the Orai1 nexus transduces the STIM1-binding signal through a conformational change in the inner core helices, and that STIM1 remotely gates the Orai1 channel without the necessity for direct STIM1 contact with the pore-forming helix.

## Abstract (web summary)

How plasma membrane Orai Ca^2+^ channels are activated by STIM proteins to activate Ca^2+^ signals is still not fully known. Here the authors show that a nexus region located at the Orai1 C-terminus allows channel gating without a direct interaction of STIM1 with the channel pore.

Ion channels transduce primary signals through gating mechanisms of extraordinary molecular precision. The widely expressed Orai family of plasma membrane (PM) Ca^2+^ entry channels are gated by the endoplasmic reticulum (ER) Ca^2+^-sensing stromal interaction molecule (STIM) proteins through a unique intermembrane conformational coupling mechanism^1^,^2^,^3^. Triggered by ER Ca^2+^ store depletion, the STIM1 ER membrane protein migrates into ER–PM junctions where it tethers and activates Orai1 channels located in the PM. The opened Orai1 channel mediates ‘store-operated' Ca^2+^ entry signals, which are critical in controlling gene expression, growth, secretory and motile responses in almost all cell types. Changes in the operation of Orai1-mediated signals are implicated in a spectrum of immunological, muscular and inflammatory disease states^2^,^4^,^5^,^6^.

Despite intense study, the molecular nature of the STIM1–Orai1 coupling interface and the mechanism of Orai1 channel activation have remained obscure. A strong binding site for STIM1 exists on the short cytoplasmic C-terminal domain of Orai1 (refs 7, 8, 9). This site lies at the periphery of the hexameric channel structure, distant from the central N-terminal pore-forming helices. Numerous studies have suggested that STIM1 simultaneously binds to both the Orai1 C-terminal and N-terminal pore itself to induce channel gating^10^,^11^,^12^,^13^. Here we reveal that a discrete five-amino-acid sequence in Orai1 creates a critical nexus between the peripheral C-terminal STIM1-binding site and the inner core helices surrounding the central N-terminal pore. The nexus comprises a flexible ‘hinge' and hydrophobic ‘hinge plate' attaching it to the channel body. Mutation of the nexus transforms the Orai1 channel into a persistently open state, indistinguishable from the STIM1-activated state. Our studies militate against the widely held two-site gating model involving direct STIM1 binding to the N-terminal pore-forming helix to open the channel^7^,^9^,^10^,^11^,^12^,^13^,^14^,^15^,^16^,^17^. Instead, we present evidence that the nexus functions as a STIM1-triggered conformational switch that ‘remotely controls' Orai1 channel gating through internal helical interactions leading to opening of the pore mouth.

## Results

### Mutation of the Orai1 nexus constitutively opens the channel

**Figure 1:**
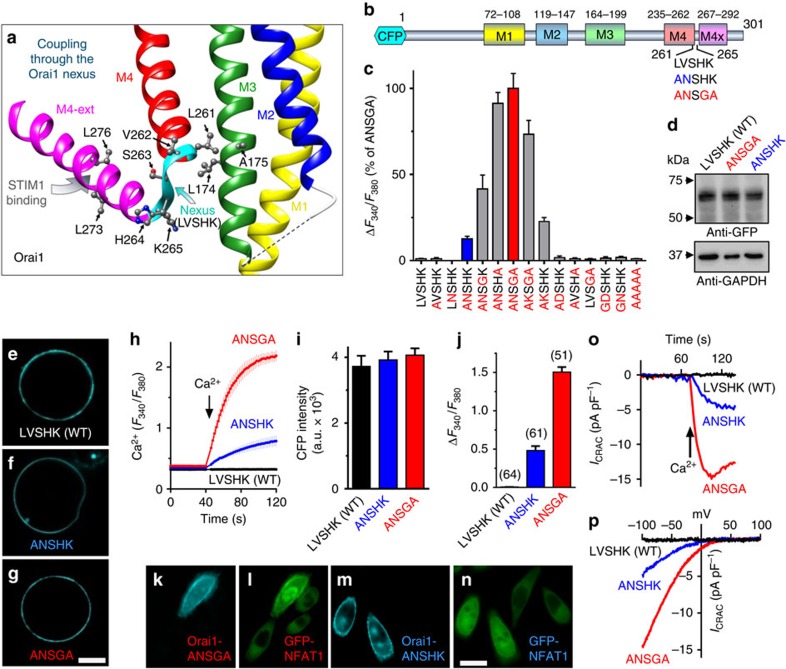
The Orai1-ANSGA nexus mutation mediates constitutive store-independent CRAC channel activity. (**a**) Schematic representation of the human Orai1 nexus (LVSHK; 261–265) and surrounding helices (Supplementary Fig. 1). (**b**) Diagram of hOrai1 transmembrane -helices and nexus mutations. (**c**) Peak constitutive Ca^2+^ entry mediated by CFP-Orai1 nexus mutations expressed in HEK cells, compared with the effect of ANSGA (100%). (**d**) Expression of CFP-Orai1-LVSHK (WT), CFP-Orai1-ANSGA and CFP-Orai1-ANSHK detected with GFP antibody, compared with GAPDH expression. (**e**–**g**) PM localization of CFP-Orai1-LVSHK (WT), CFP-Orai1-ANSHK and CFP-Orai1-ANSGA in HEK cells. Scale bar, 5 μm (**h**) Fura-2 ratiometric constitutive Ca^2+^ responses in HEK cells expressing CFP-Orai1-LVSHK (WT), CFP-Orai1-ANSHK or CFP-Orai1-ANSGA. (**i**) CFP fluorescence intensity (a.u.) of cells in **h**. (**j**) Average peak Ca^2+^ entry responses from three independent experiments. (**k**–**n**) Nuclear translocation of NFAT-GFP (**l**,**n**) induced by CFP-Orai1-ANSGA (**k**,**l**) or CFP-Orai1-ANSHK (**m**,**n**) transfected into Hela cells stably expressing NFAT-GFP. Note, all cells express NFAT-GFP, but only one cell (**k**,**l**) or two cells (**m**,**n**) are transfected with Orai1 nexus mutants, respectively. Scale bar, 10 μm (**o**) Whole-cell *I*_CRAC_ in store-replete HEK cells transfected with CFP-Orai1-WT, CFP-Orai1-ANSHK or CFP-Orai1-ANSGA. (**p**) *I*–*V* relationship for cells in **o**. All values are means±s.e.m.

The recently solved *Drosophila* Orai structure reveals the four-transmembrane spanning protein forms a hexameric channel (Supplementary Fig. 1)^18^. Highly conserved and with nearly identical transmembrane helices, the human Orai1 channel has a central ring of pore-forming M1 transmembrane helices that are packed tightly against the M2 and M3 transmembrane helices (Fig. 1a and Supplementary Fig. 1a)^2^,^18^. The M4 transmembrane helix lies at the outer periphery and has a cytoplasmic extension (M4-ext), which provides the strong binding site for STIM1 (Fig. 1a)^7^,^9^,^18^,^19^. The C-terminal M4-ext is connected to M4 by a conserved flexible ‘hinge' (SHK; residues S263, H264 and K265)^13^,^18^,^20^. Immediately upstream of the hinge, residues V262 and L261 closely approach the M3 helix, with L261 in close contact with L174 and A175 (Fig. 1a). We define the 261–265 sequence (LVSHK: L261, V262, S263, H264 and K265) as the ‘nexus' because it is the first point of close contact between the STIM1-binding M4-ext and the cluster M3/M2/M1 helices forming the channel core.

**Figure 2:**
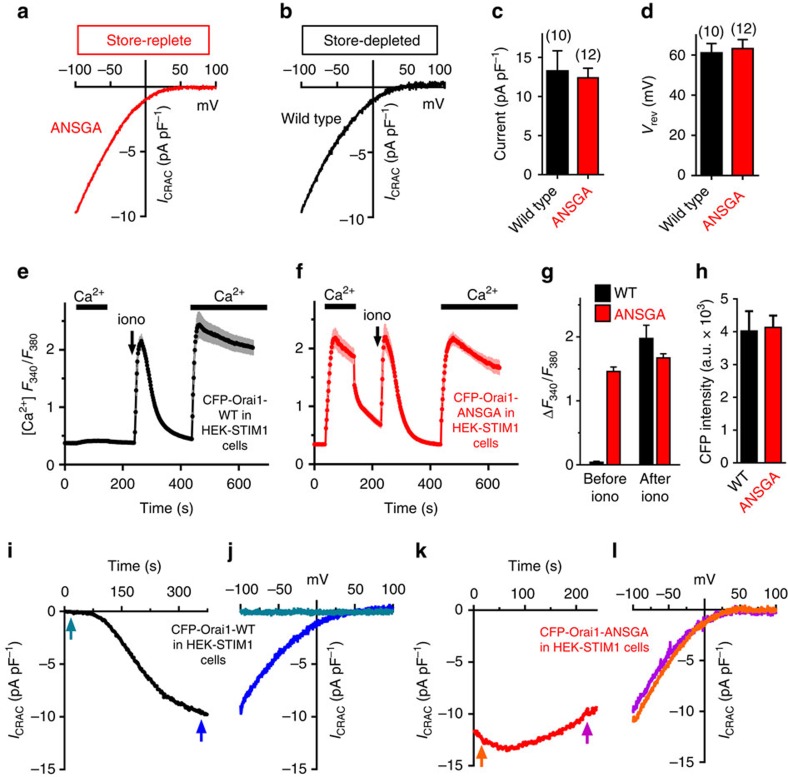
The Orai1-ANSGA mutant is fully active and independent of STIM1. (**a**) *I*–*V* relationship in store-replete HEK cells expressing CFP-Orai1-ANSGA. (**b**) *I*–*V* relationship in store-depleted HEK cells expressing CFP-Orai1-WT and STIM1-YFP. (**c**) Summary of the peak current amplitude at −100 mV. (**d**) Summary of the reversal potential measured from *I*–*V* data for Orai1-WT activated by store depletion and constitutively active Orai1-ANSGA in store-replete cells. (**e**,**f**) Fura-2 Ca^2+^ responses in stable HEK-STIM1-YFP cells expressing CFP-Orai1-WT (**e**) or CFP-Orai1-ANSGA (**f**) before and after store depletion with 2.5 μM ionomycin. (**g**) Summary of the constitutive (before ionomycin) and store-dependent (after ionomycin) Ca^2+^ entry in cells shown in **e**,**f**. (**h**) CFP fluorescence intensity of cells in **e**,**f**. (**i**,**j**) Internal BAPTA dialysis to deplete stores caused *I*_CRAC_ to develop over time in HEK-STIM1-YFP cells expressing CFP-Orai1-WT. (**k**,**l**) With identical BAPTA-treated HEK-STIM1-YFP cells expressing CFP-Orai1-ANSGA, maximal *I*_CRAC_ was observed at break-in. *I*–*V* curves (**j**,**l**) were determined at arrows shown (**i**,**k**). All values are means±s.e.m.

Expressed in human embryonic kidney (HEK) cells, Orai1 channels with mutations in the nexus resulted in profound store-independent constitutive channel activity (Fig. 1b,c). While mutation of either L261 or V262 alone yielded no constitutive activity, the combination of L261A with either V262N or V262K resulted in substantial constitutive channel activity in store-replete HEK cells. These mutations combined with H264G and/or K265A hinge mutations produced even greater constitutive channel activation (Fig. 1b,c). The highly constitutively active Orai1-ANSGA and less-active Orai1-ANSHK channel derivatives (Fig. 1c) were expressed in HEK cells similarly to wild-type (WT) Orai1-LVSHK (Fig. 1d) and were exclusively localized to the PM (Fig. 1e–g). The constitutive activity of Orai1-ANSGA or Orai1-ANSHK contrasted with the complete lack of Ca^2+^ entry of identically expressed Orai1-WT (LVSHK) (Fig. 1h–j). Ca^2+^ entering through the Orai1 channel is known to specifically activate the transcription factor, nuclear factor of activated T cells (NFAT)^2^,^4^,^5^,^6^. Constitutive Ca^2+^ entry mediated by Orai1-ANSGA or Orai1-ANSHK fully activated the translocation of green fluorescent protein tagged NFAT1 (GFP-NFAT1) into the nucleus (Fig. 1k–n), whereas untransfected cells in the same image field showed no GFP-NFAT1 translocation. Orai1-ANSGA or Orai1-ANSHK expressed in HEK cells gave large constitutive Ca^2+^ inward currents (Fig. 1o). In contrast, with identical pipette solutions, STIM1 stably transfected HEK cells (HEK-STIM1 cells) expressing WT Orai1 showed no inward current (Supplementary Fig. 2), hence no store depletion occurred under this condition. For both mutants, the *I*/*V* relationship revealed inward rectification typical of the Ca^2+^-release-activated Ca^2+^ (CRAC) current (*I*_CRAC_) (Fig. 1p), which is the signature of Orai1 channels^2^. The current in store-replete HEK cells expressing Orai1-ANSGA (without STIM1 overexpression; Fig. 2a) was similar to that in HEK cells expressing both Orai1-WT and STIM1 after store depletion (Fig. 2b,c) and, most notably, the reversal potential in each case was the same (Fig. 2d). The results reveal that the Orai1-ANSGA mutant mediates authentic, constitutive *I*_CRAC_, indistinguishable from CRAC current mediated by Orai1-WT in response to STIM1.

### Orai1-ANSGA is fully active and STIM1-independent

Important to test was whether the Orai1-ANSGA mutant was fully active or if store depletion could further increase Ca^2+^ entry. Orai1-WT expressed in HEK-STIM1 cells has no constitutive function, but is fully activated after ionomycin-induced store depletion (Fig. 2e). Orai1-ANSGA expressed in HEK-STIM1 cells gave large constitutive Ca^2+^ entry before store depletion with similar rate and almost the same peak as that seen after store depletion (Fig. 2f–h). We also compared the onset of *I*_CRAC_ with either Orai1-WT (Fig. 2i,j) or Orai1-ANSGA (Fig. 2k,l) following store depletion on addition of BAPTA. Development of *I*_CRAC_ with Orai-WT was slow reflecting the rate of store depletion (Fig. 2i). In contrast, Orai1-ANSGA already had maximal constitutive *I*_CRAC_ at break-in and there was no additional current developed after store depletion (Fig. 2k). The *I*/*V* relationship revealed the inward rectifying *I*_CRAC_ properties remained unchanged before and after store depletion (Fig. 2l).

### The activated Orai1 nexus mutant alters STIM1 binding

**Figure 3:**
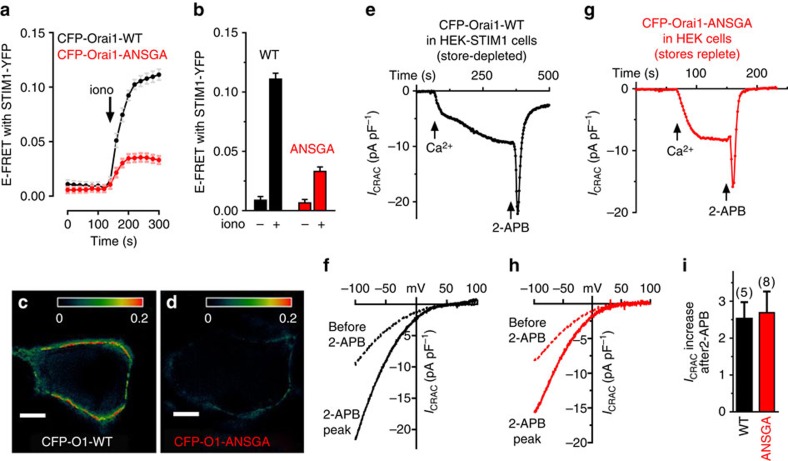
The Orai1-ANSGA mutant has modified STIM1 binding but is pharmacologically identical to the STIM1-activated Orai1-WT. (**a**) E-FRET changes in HEK-STIM1-YFP cells transiently expressing CFP-Orai1-WT (black) or CFP-Orai1-ANSGA (red) following ER Ca^2+^ store depletion with ionomycin (2.5 μM). (**b**) Summary of E-FRET values for the Orai1 mutants-WT and mutant before and after store depletion. Calculated FRET image from CFP-Orai1-WT (**c**) and CFP-Orai1-ANSGA (**d**) 3 min after ionomycin. Scale bar, 5μm. (**e**,**f**) Using internal BAPTA-dialysed HEK-STIM1-YFP cells expressing CFP-Orai1-WT, *I*_CRAC_ was observed on 20 mM external Ca^2+^ addition. A unit of 50 μM 2-APB induced rapid transient *I*_CRAC_ activation (*I*–*V* curves shown were 8 s before 2-APB, and at the peak following 2-APB addition). (**g**,**h**) In store-replete HEK cells expressing CFP-Orai1-ANSGA, constitutive *I*_CRAC_ was measured on external Ca^2+^ addition, and 50 μM 2-APB added as in **e** giving identical, rapid transient activation of *I*_CRAC_, as revealed from *I*–*V* curves 8 s before 2-APB, and at the peak following 2-APB addition. (**i**) Summary of 2-APB induced increase in *I*_CRAC_ from individual cells. All values are means±s.e.m.

Since modification of the Orai1 STIM1-binding site nexus to ANSGA functionally mimics STIM1 binding, we investigated whether this opened channel configuration altered STIM1 binding. Indeed, the ANSGA mutation substantially decreased E-Förster resonance energy transfer (FRET) between yellow fluorescent protein-tagged STIM1 (STIM1-YFP) and cyan fluorescent protein-tagged Orai1 (CFP-Orai1) (Fig. 3a–d). From the crystal structure, the C-terminal Orai1 M4-ext STIM1-binding sequences are hydrophobically linked between adjacent Orai1 subunit pairs in the closed channel state^18^ (Supplementary Fig. 1). It is shown that STIM1-binding induces rearrangement of these adjacent, conjoined M4-ext pairs^11^,^20^,^21^. The Orai1-ANSGA open-state channel mutant may undergo a similar rearrangement of M4-ext STIM1-binding pairs that may now no longer be optimal for binding STIM1. Earlier studies by Navarro-Borelly *et al*.^21^ measuring Orai1–Orai1 interactions by FRET, revealed that STIM1 binding triggers a molecular rearrangement of the Orai1 C terminus resulting in a decrease in FRET between Orai1 subunits. Expressing C-terminally tagged CFP- and YFP-Orai1 mixtures, we observed that store depletion induced a much smaller change in Orai–Orai FRET for Orai1-ANSGA compared with Orai1-WT (Supplementary Fig. 3a–c), consistent with ANSGA already being in an ‘active' state. A model depicting such a scheme is shown in Supplementary Fig. 3d,e)

### The active Orai1-ANSGA mutant is pharmacologically unchanged

The authenticity of the opened state of the Orai1-ANSGA mutant is further underscored by actions of 2-aminoethoxy-diphenylborane (2-APB). *I*_CRAC_ mediated by Orai1-WT in response to store depletion undergoes a characteristic biphasic response to 50 μM 2-APB (Fig. 3e,f), typical of that observed in numerous prior studies^22^,^23^. Constitutive *I*_CRAC_ mediated by Orai-ANSGA responded almost identically (Fig. 3g,h). Indeed, the degree of potentiation of current by 2-APB was almost identical between store-activated Orai1-WT and constitutively active Orai1-ANSGA (Fig. 3i). The complex action of 2-APB on Orai1 channel activation was thought to reflect changes in STIM1 association with Orai1, as well as direct channel effects^24^. The identical 2-APB action on Orai1-WT and Orai1-ANSGA suggests 2-APB targets the gated channel. Thus, the STIM1-independent Orai1-ANSGA open channel may provide a useful tool for discerning mechanism of action of 2-APB.

### Orai1 M4–M3 helix coupling transduces STIM1-induced gating

**Figure 4:**
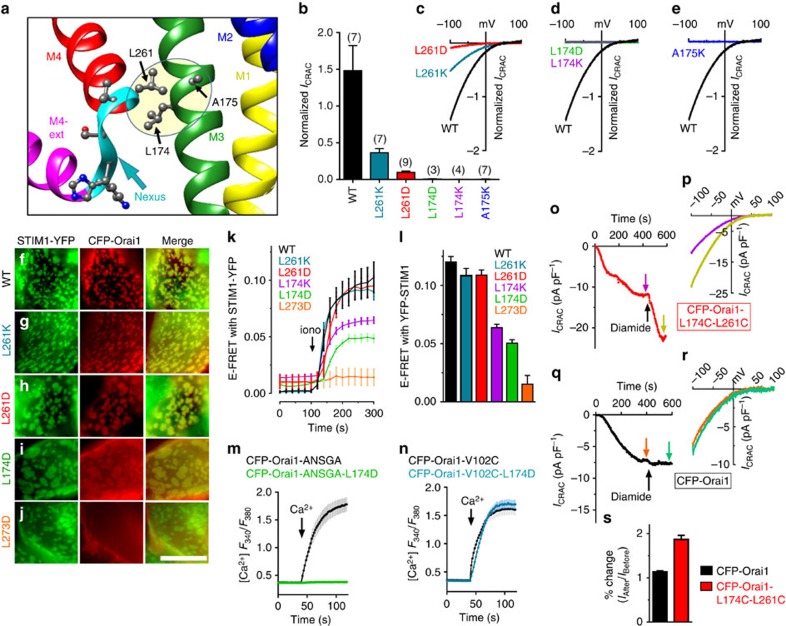
A critical M4–M3 interaction within Orai1 transduces the binding of STIM1 into channel gating. (**a**) Detail of the M4 (L261) interaction with M3 (L174 and A175) shown as the shaded area. (**b**) Normalized peak currents for L261, L174 and A175 CFP-Orai1 mutants, expressed in stable HEK-STIM1-YFP cells, store-depleted with 10 mM BAPTA internal solution. *I*_CRAC_ measured in 20 mM Ca^2+^ at −100 mV was normalized for each individual cell to the fluorescence intensity of CFP-Orai1 mutant expression in that cell. (**c**–**e**) Representative *I*–*V* plots of WT and mutant Orai1 channels. (**f**–**j**) High-resolution imaging of the ER–PM interface in HEK-STIM1-YFP cells transiently expressing WT (**f**), L261K (**g**), L261D (**h**), L174D (**i**) and L273D (**j**), 3 min after 2.5 μM ionomycin treatment. Scale bar, 5 μm. (**k**) E-FRET between STIM1-YFP and CFP-Orai1 using the same Orai1 mutants expressed within HEK-STIM1-YFP cells as in **b**. (**l**) Summary of E-FRET peak values after store depletion for the Orai1 mutants shown for **k**. (**m**) Fura-2 Ca^2+^ responses in store-replete HEK cells expressing CFP-Orai1-ANSGA (black) or CFP-Orai1-ANSGA-L174D (green). (**n**) Fura-2 Ca^2+^ responses in store-replete HEK cells expressing CFP-Orai1-V102C (black) or CFP-Orai1-V102C-L174D (blue). (**o**–**r**) Whole-cell recordings of stable HEK-STIM1-YFP cells expressing CFP-Orai1-L174C/L261C (**o**,**p**) or CFP-Orai1 (**q**,**r**). ER stores were depleted with 10 mM internal BAPTA. A unit of 500 μM diamide was applied (arrow) after *I*_CRAC_ development. *I*–*V* plots (**p**,**r**) are shown for current before and after diamide as shown in **o**,**q** (arrows). (**s**) Summary of diamide-induced current enhancement in **o**,**q**. All values are means±s.e.m.

Our studies reveal that a discrete M4–M3 interacting site within Orai1 is a crucial locus mediating the remote allosteric gating of the channel. On the basis of the dOrai crystal structure, L261 in human Orai1 at the proximal end of the M4-nexus is closely juxtaposed with L174 and A175 on the M3 helix (Fig. 4a). To investigate whether possible hydrophobic interactions between these M4 and M3 side chains might transduce the STIM1-binding signal, we mutated these residues in Orai1-WT to polar or charged side chains to disrupt their interactions (Fig. 4b–e). Orai1 L261K or L261D mutations substantially reduced store-dependent *I*_CRAC_ with little alteration in PM expression (Supplementary Fig. 4). The L261 residue is conserved across most species and Orai subtypes, and appears to play an important role in mediating STIM1-induced channel activation. The critical M4–M3 coupling role is yet further revealed by mutating the adjacent M3 nonpolar L174 or A175 residues with charged residues (D or K), which in each case entirely eliminated current. These marked effects on Orai1 functional coupling to STIM1 prompted us to examine how interference in the M4–M3 interaction alters STIM1 binding. None of the mutations in L261 or L174 prevented association of Orai1 with punctal STIM1 following store depletion (Fig. 4f–i), in stark contrast to the Orai1-L273D mutation known to entirely block STIM1 binding^8^,^11^ (Fig. 4j). FRET analysis of STIM1-YFP and CFP-Orai1 mutant interactions provided important further information (Fig. 4k,l). While the CFP-Orai1-L273D mutation abolished FRET with STIM1-YFP as expected, the L174K or L174D mutants showed substantially reduced FRET suggesting interference of the M4–M3 interaction could alter the STIM1-binding site. However, the L261K or L261D mutations had no effect on FRET with STIM1. Thus, the M4–M3 connection appears to be crucial for transducing the STIM1-binding signal into channel gating, and can be severed without necessarily affecting STIM1 binding. These results suggest that the STIM1-induced gating of Orai1 is exclusively mediated by the M4–M3 connection and that interactions of STIM1 with other ‘gating' sites on the Orai1 channel are unlikely. We provide more compelling evidence for this later.

A crucial experiment was to assess how the L174D mutation altered the constitutively active Orai1-ANSGA mutant. The complete blocking effect of L174D (Fig. 4m) further reinforced the identity between the gating mechanism of Orai1-ANSGA and that of STIM1-induced Orai1-WT. Interestingly, in dOrai1, the M3-residue equivalent to L174 is T246. Mutating L174 to threonine in human Orai1 did not affect STIM1 association but modestly reduced current (Supplementary Fig. 5), suggesting that tuning of the efficacy of STIM1-induced gating may be a significant evolutionary factor. Importantly, the L174D mutation was without any effect on the powerful constitutively active Orai1-V102C pore mutation (Fig. 4n). This mutation is in the hydrophobic cleft of the Orai1 pore itself and renders the channel constitutively open but with considerably altered cation selectivity^25^. The V102C mutant changes only the pore itself (not the physiological gating mechanism), and the lack of action of L174D on its function underscores the crucial distinction between this open-pore mutant and WT-Orai1 activated by either STIM1 or the ANSGA mutation.

Yet further evidence that M4–M3 interactions mediate Orai1 gating was derived from crosslinking experiments. Mutating the apposed L174 and L261 residues in Orai1-WT to cysteines gave a functional channel still activated by store depletion (Fig. 4o,p). Addition of diamide to the double cysteine Orai1 mutant caused a rapid and substantial increase in current (Fig. 4o,s) without alteration of selectivity (Fig. 4p). In contrast, the diamide addition did not alter the Orai1-WT channel activity (Fig. 4q–s). We also examined whether the increased CRAC current observed after diamide-induced crosslinking might reflect an increase in the interaction of STIM1 with Orai1. Hence, we examined FRET between STIM1-YFP and CFP-Orai1 (or Orai1-WT and Orai1-L174C-L261C) before and after diamide addition. As shown in Supplementary Fig. 6, there was significant change in STIM-Orai FRET following diamide addition. Thus, disulfide-mediated M4–M3 crosslinking enhances STIM1-mediated channel gating, reinforcing the strategic importance of the M4–M3 interaction in STIM1-induced channel gating.

### The Orai1-ASNGA channel remains open without STIM1 binding

**Figure 5:**
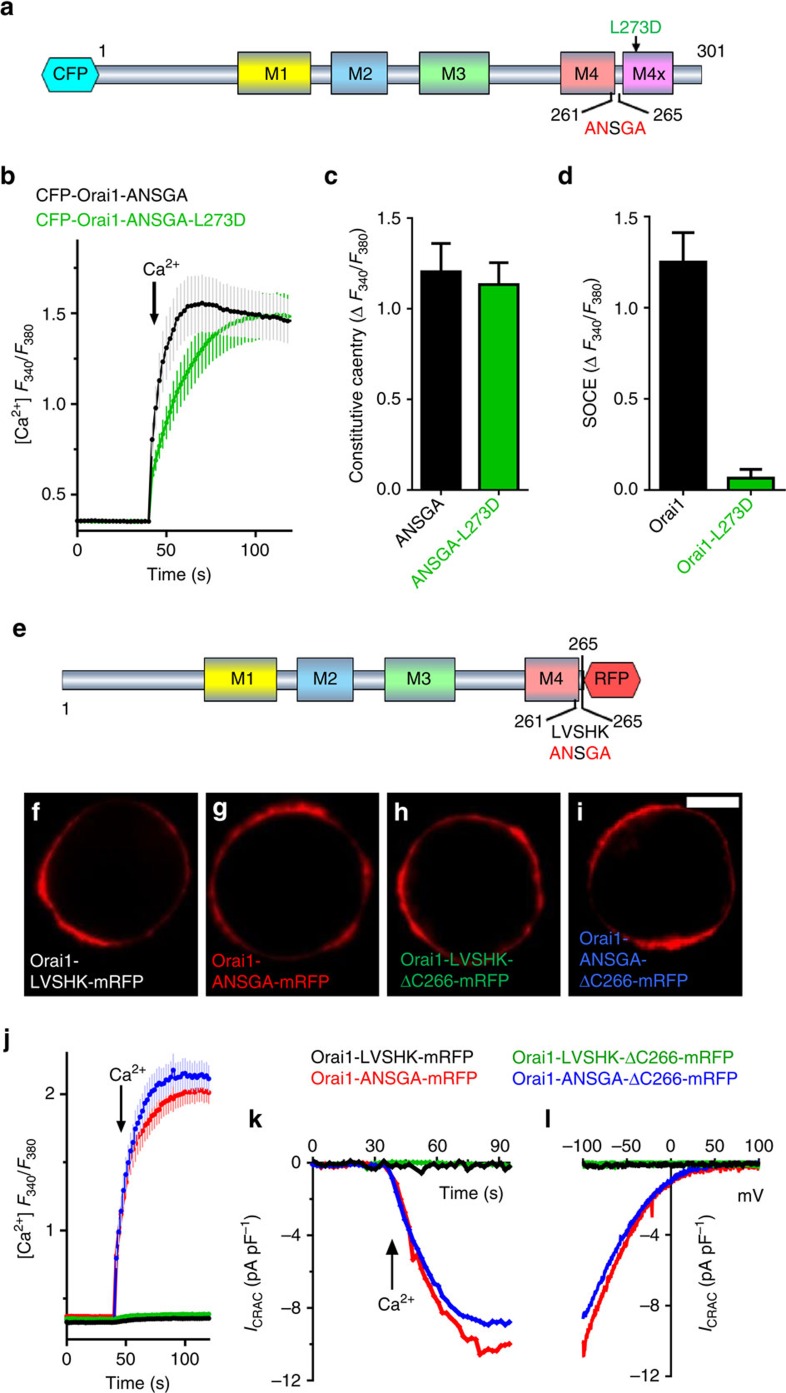
The open Orai1-ASNGA channel is independent of the C-terminal STIM1-binding site. (**a**) The CFP-Orai1-ANSGA-L273D construct. (**b**) Constitutive fura-2 Ca^2+^ entry responses in store-replete HEK cells expressing CFP-Orai1-ANSGA (black) or CFP-Orai1-ANSGA-L273D (green). (**c**) Summary of the effect of the L273D mutation on constitutive Ca^2+^ entry mediated by CFP-Orai1-ANSGA. (**d**) Summary of the effect of L273D mutation on store-operated Ca^2+^ entry mediated by CFP-Orai1-WT in HEK-STIM1-YFP cells after ionomycin-induced store depletion. (**e**) The Orai1-LVSHK-ΔC266-mRFP and Orai1-ANSGA-ΔC266-mRFP truncation constructs. (**f**–**i**) PM localization of the mRFP-tagged Orai1-WT or Orai1-ANSGA mutant and their ΔC266 truncations expressed in HEK cells. Scale bar, 5 nm. (**j**) Constitutive fura-2 Ca^2+^ entry responses in store-replete HEK cells expressing the mutations in **f**–**i**. (**k**) Whole-cell recordings for store-replete HEK cells expressing the mutations in **j**. (**l**) *I*–*V* curves from the same cells as **k**. All values are means±s.e.m.

The Orai1-ANSGA construct had important utility in allowing us to definitively assess the roles of the cytoplasmic Orai1 N and C termini in Orai1 channel gating. First, we examined whether the Orai1 C-terminal STIM1-binding site played any role in the constitutively active Orai1-ANSGA channel, perhaps through endogenous STIM proteins. Mutation of Orai1 L273 to D completely prevented STIM1 binding^8^,^11^, but did not prevent Orai1-ANSGA-mediated constitutive Ca^2+^ entry while completely blocking Orai1-WT function (Fig. 5a–d). More significantly, we truncated the Orai1 channel cytoplasmic C terminus to remove the entire STIM1-binding site. Since such truncation can prevent Orai1 PM insertion^7^,^26^, we added monomeric red fluorescent protein (mRFP) to the Orai1 C terminus (Fig. 5e). We compared function of either full-length Orai1 or constructs with residues 266–301 deleted (Δ266) resulting in C-termini ending in the nexus residues (261–265), either LVSHK (WT) or ANSGA. All four constructs were exclusively expressed in the PM (Fig. 5f–i). The truncated Orai1-Δ266-ANSGA-mRFP construct gave constitutive Ca^2+^ entry and *I*_CRAC_ almost identical to that of the full-length Orai1-ANSGA-mRFP construct (Fig. 5j–l). In contrast, neither truncated nor full-length Orai1 versions containing the WT nexus (LVSHK) showed any constitutive entry (Fig. 5j–l). This provides compelling evidence that the action of Orai1-ANSGA is independent of any STIM1 input.

### Role of the Orai1 N terminus in STIM1-induced channel gating

**Figure 6:**
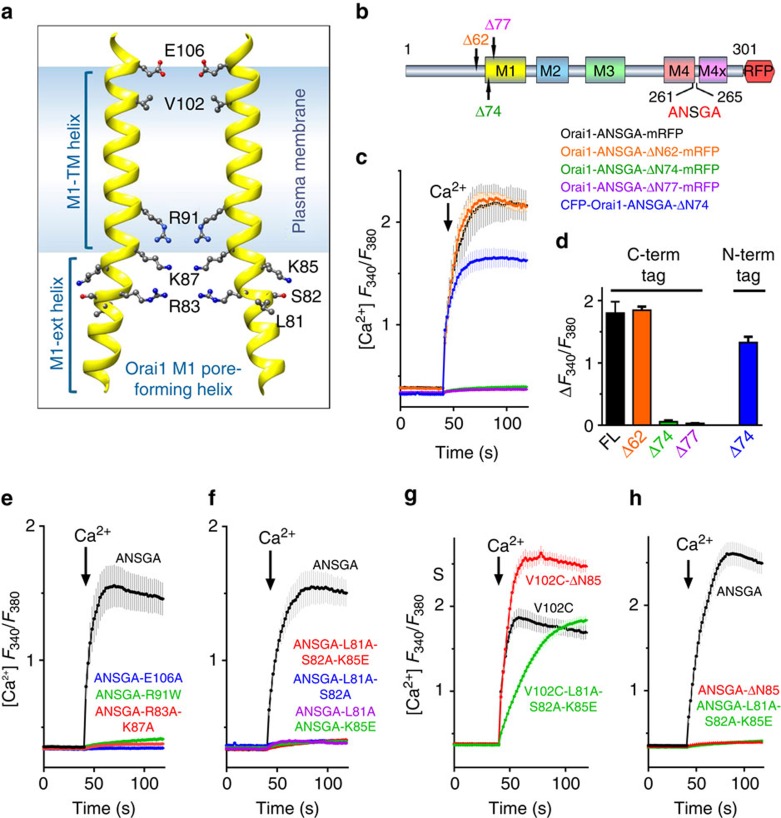
Disruption of the Orai1 N-terminal M1-ext compromises pore function but is not the STIM1 gating site. (**a**) The pore-forming M1 helices of two opposing subunits of the hexameric Orai1 channel showing key residues to be mutated. (**b**) N-terminal truncations (Δ62, Δ74 and Δ77) of Orai1-ANSGA-mRFP. (**c**) Constitutive fura-2 Ca^2+^ responses in HEK cells expressing Orai1-ANSGA-mRFP (black), Orai1-ANSGA-ΔN62-mRFP (orange), Orai1-ANSGA-ΔN74-mRFP (green), Orai1-ANSGA-ΔN77-mRFP (purple) or CFP-Orai1-ANSGA-ΔN74 (blue). (**d**) Summary of the peak Ca^2+^ entry in **c**. (**e**–**h**) Constitutive fura-2 Ca^2+^ entry responses in store-replete HEK cells expressing the Orai1 mutants shown: (**e**) key Orai1-M1 pore-lining mutations in CFP-Orai1-ANSGA; (**f**) purported STIM1-binding Orai1-M1-ext outward-facing mutations in CFP-Orai1-ANSGA; (**g**) Orai1-M1-ext mutations or Δ1–85 truncation in CFP-Orai1-V102C; (**h**) Orai1-M1-ext mutations or Δ1–85 truncation in CFP-Orai1-ANSGA. All values are mean±s.e.m.

Finally, we addressed the role of the Orai1 N terminus in channel activation. While the cytoplasmic N-terminal residues 1–72 are redundant for channel function^7^,^9^,^11^,^16^, the subsequent 73–86 sequence forms a four helical-turn cytoplasmic M1 extension (M1-ext) to the M1 pore (Fig. 6a)^17^,^18^. This M1-ext region is indispensable for channel function^9^,^11^,^14^,^15^,^16^. Considerable evidence has suggested that the Orai1 M1-ext plays an active role in STIM1-induced gating of the Orai1 channel^7^,^9^,^10^,^11^,^12^,^13^,^14^,^15^,^16^,^17^,^26^. It has been shown that STIM1 undergoes weak interactions with the M1-ext^9^,^14^, and that M1-ext mutations can lower FRET between STIM1 and Orai1 (refs 11, 16). Recently, an Orai1 M1-ext peptide (66–91) was shown to interact with STIM1 and block STIM1-mediated Orai1 activation^12^. However, despite the apparent congruity among these studies, conclusive proof that STIM1 binding to the Orai1 N terminus is required for channel opening has not been forthcoming^2^.

The STIM1-independent Orai1-ANSGA open channel provides a definitive tool to probe the crucial question of whether STIM1 binding to the Orai1 N terminus is necessary to gate the channel. As expected, N-truncation of the first 62 N-terminal residues from Orai1-ANSGA-mRFP (Δ62) was without effect, whereas truncation of the first 74 or 77 N-terminal residues (Δ74 and Δ77), almost or completely eliminated Ca^2+^ entry, respectively (Fig. 6b–d). This is largely in agreement with the inhibitory effects of N-terminal truncation on STIM1-induced activation of WT Orai1 (refs 7, 9, 11, 16). However, we noted that the complete loss of Ca^2+^ entry with the Orai1-ANSGA (Δ74) construct was distinct from the partial effect of STIM1-activated Orai1 (Δ74) described earlier^11^,^16^ reflecting that this deletion is on the cusp of the truncation effect. Since the former results were obtained using N-terminally CFP-tagged Orai1 truncations, we examined N-terminally CFP-tagged CFP-Orai1-ANSGA (Δ74) and observed an identical partial effect (Fig. 6c,d, blue trace). Thus, the results reveal complete identity in the effects of N-terminal truncation on STIM1-induced Orai1 and the constitutively active Orai1-ANSGA.

Since Orai1-ANSGA is open without STIM1, this indicates that the Orai1 M1-ext terminus is required for maintaining channel integrity as opposed to functioning as the locus for STIM1-induced channel gating. We also examined certain well-defined Orai1 pore-lining M1-helix mutations on Orai1-ANSGA function, including E106A, R91W, K87A and R83A. All these pore mutations completely prevented Orai1-ANSGA function (Fig. 6e) exactly as they prevent STIM1-induced WT Orai1 channel function^2^, further establishing the authenticity of the Orai1-ANSGA constitutively gated channel. We then focused on three residues (L81, S82 and K85) oriented outward from the M1-ext helix (Fig. 6a) and recently reported as the N-terminal binding site required for STIM1 mediating Orai1 channel gating^12^. Remarkably, the triple ‘LSK' mutant (L81E–S82A–K85E) entirely prevented function of the Orai1-ANSGA mutant, as also did the double mutant L81A–S82A (Fig. 6f). Even the single-point mutations, L81A and K85E, completely prevented Orai1-ANSGA channel function. Indeed, the single L81A mutation in Orai1-WT completely blocked channel function without altering STIM1-Orai1 association or FRET (Supplementary Fig. 7). Importantly, the M1-ext LSK mutant does not simply corrupt channel function per se. Thus, neither the LSK mutation nor the Orai1-ΔN85 truncation (devoid of almost the entire M1-ext) blocked constitutive function of the V102C open-pore construct^25^ (Fig. 6g), in complete contrast to the total block of Orai1-ANSGA function (Fig. 6h). Hence, the M1-ext plays no role in the constitutively open Orai1-V102C channel, underscoring its distinction from both the Orai1-ANSGA and STIM1-activated Orai1 channels which are both equivalent.

## Discussion

Our results reveal that a key ‘nexus' segment (LVSHK) within Orai1, linking the peripheral C-terminal STIM1-binding site with the main channel body (Fig. 1a), is the critical mediator of channel gating. The Orai1-ANSGA nexus mutant activates the channel and likely mimics the exact conformational change in the C-terminal M4-ext effected by STIM1 binding. The nexus contains two components—the ‘hinge' sequence (SHK; 263–265)^13^,^18^,^20^ and what we term the ‘hinge plate' (LV; 261–262) that attaches it hydrophobically to M3. Two recent studies revealed that proline substitution to distort the SHK hinge or cysteine crosslinking to lock the resting hinge configuration, both prevent STIM1 binding to Orai1 and channel activation^13^,^20^. However, neither study examined the adjacent, more critical LV ‘hinge-plate' residues. L261 likely undergoes hydrophobic coupling with L174 (and A175) in the M3 helix to gate the Orai1 channel, an interaction that we show can be blocked by charged residue replacement or enhanced by cysteine crosslinking. The decreased STIM1 binding observed for the constitutively active Orai1-ANSGA construct likely reflects that the M4-ext segments critical for forming the STIM1-binding site are reconfigured in such a manner that they are no longer in the optimal STIM1-binding configuration. Thus, mutation of the nexus reflects the intimate connection between STIM1 binding and channel activation.

**Figure 7:**
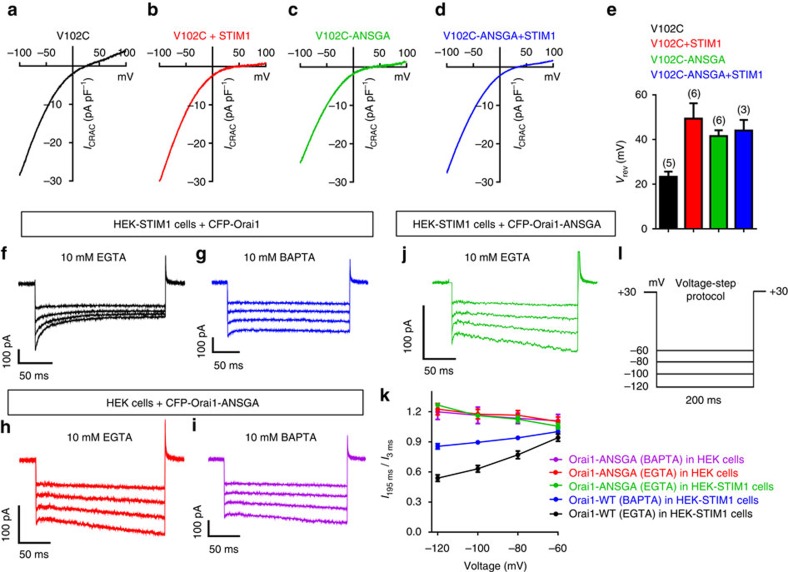
The Orai-ANSGA mutation restores ion selectivity to the Orai1-V102C mutant and exhibits no CDI. (**a**) Current–voltage relationship for Orai1-V102C expressed in HEK cells (without STIM1 expression) with cytosolic Ca^2+^ buffered to 90 nM with EGTA (stores replete), revealing a reversal potential of ∼25 mV reflecting the decreased Ca^2+^ selectivity of the pore mutant. (**b**) *I*–*V* for Orai1-V102C expressed in HEK-STIM1 cells with 10 mM BAPTA in the pipette solution to deplete Ca^2+^ stores, revealing right-shifted reversal potential similar to WT Orai1. (**c**) *I*–*V* for the Orai1-V102C-ANSGA construct expressed in HEK cells without expressed STIM1 with Ca^2+^ buffered to 90 nM (stores replete) as in **a**, revealing that the reversal potential is shifted right-ward similar to the shift induced by STIM1 and store emptying. (**d**) *I*–*V* for the Orai1-V102C-ANSGA expressed in HEK-STIM1 cells with 10 mM BAPTA-induced store emptying, revealing that the right shift by ANSGA is not further altered by STIM1. (**e**) Summary of the reverse potential data shown in **a**–**d**. Values are means±s.e.m. of number of individual cells shown. (**f**,**g**) Representative CRAC currents elicited by 200 ms hyperpolarization in HEK-STIM1-YFP cells expressing CFP-Orai1 with 10 mM EGTA (**f**) or 10 mM BAPTA (**g**) in the pipette solution. (**h**,**i**) The same CRAC current measurements undertaken with HEK cells expressing CFP-Orai1-ANSGA with 10 mM EGTA (**h**) or 10 mM BAPTA (**i**) in the pipette solution. (**j**) CRAC current measurements undertaken with HEK-STIM1 cells co-expressing CFP-Orai1-ANSGA with 10 mM EGTA in the pipette solution. (**k**) Summary of quantified CDI defined as the current recorded at 185 ms divided by the current recorded at 2 ms after each pulse. Orai1-WT (EGTA), *n*=6; Orai1-WT (BAPTA), *n*=5; Orai1-ANSGA (EGTA), *n*=8; Orai1-ANSGA (BAPTA), *n*=7. All values are means±s.e.m. (**l**) Protocol for CDI measurement: after maximum current was reached, holding potential steps from +30 to −60, −80, −100 and −120 mV were applied with 5 s intervals between steps. The currents were recorded in 20 mM Ca^2+^ Ringer's solution.

We hypothesize that the key M4–M3 coupling trigger is propagated through the tightly associated M3, M2 and pore-forming M1 helices into a conformational alteration of critical pore residues. Important recent experiments measuring Tb^3+^ luminescence in purified recombinant Orai1, reveal that STIM1 binding to the Orai1 cytoplasmic face elicits a conformational change close to the external pore entrance, detected at the Ca^2+^-selectivity filter residue, E106, and at the adjacent pore-lining residue, V102 (ref. 12). This supported the key earlier finding that STIM1 binding can induce alteration of the ion-selectivity of the constitutively open V102C mutant of Orai1 (ref. 25). If the Orai1-ANSGA mutation truly mimics this transfer of information from the STIM1-binding site to the pore itself, we expect to see a similar alteration of the Orai1-V102C mutant. Indeed, as shown in Fig. 7a–e, the STIM1-induced right-shift of the reversal potential of Orai1-V102C is almost the same as the shift in reversal potential induced by the ANSGA mutation. Clearly, the ANSGA mutation is transferring the same conformational change as STIM1. However, in contrast to the interpretation of Gudlur *et al*.^12^, we infer that this information is not mediated by STIM1's interaction with the Orai1 N-terminal M1-ext cytoplasmic pore face, but instead is propagated remotely from the Orai1 C-terminal M4-ext through the channel core helices. Since the STIM1-induced change in V102C channel selectivity was prevented by M1-ext mutations^11^,^16^ we cannot exclude the possibility that the M1-ext plays a role in mediating Ca^2+^ selectivity of the pore.

Interestingly, the C-terminal Orai1 mutant P245L, straightening a proline bend in the M4 helix (Supplementary Fig. 1), was recently shown to partially induce channel opening, albeit with altered characteristics^13^,^27^. Notably, we found the active Orai1-P245L mutant was completely blocked by the L174D mutation (Supplementary Fig. 8), indicating that M4 straightening-induced channel opening works through the same M4–M3 coupling mechanism as Orai1-ANSGA, and propagated through the same conformational change in the channel core helices.

Our results provide a new and simpler view of Orai1 channel gating. The identical Orai1 N-terminal structural requirements for gating of both STIM1-dependent Orai1-WT and STIM1-independent Orai1-ANSGA, indicate that the STIM1 interaction with the C-terminal Orai1-M4-ext is necessary and sufficient for channel gating. We suggest that an interaction of STIM1 with the Orai1 N terminus is not required for channel gating. This militates against a considerable body of evidence postulating that weak STIM1 interactions with the Orai N terminus mediate channel gating and/or altered ion selectivity^7^,^9^,^10^,^11^,^12^,^13^,^14^,^15^,^16^,^17^,^26^. Clearly, the necessity for N-terminal interaction with STIM1 for gating had remained uncertain^2^, and recent evidence indicated that the Orai1 C terminus does more than merely tether STIM1 and is at least involved in the gating process^11^. Of course, the entire pore-forming N-terminal M1/M1-ext helix is vital for channel function, and we suggest that M1-ext mutants and competing peptide fragments disrupt the pore function rather than alter STIM1 interaction. Thus, expression of the very peptides that form the essential and exposed M1-ext outer pore helices may ‘chelate' within the pore structure itself and hence interfere with pore function as observed^12^.

Does the reported weak binding of STIM1 to the Orai1 N terminus^9^,^14^ have functional significance? Ca^2+^-dependent inhibition (CDI) of Orai1 was shown recently to involve functional coupling between STIM1 and the Orai1 M1-ext residues W76 and Y80 (refs 28, 29). Significantly, constitutive Orai1-ANSGA-mediated *I*_CRAC_ is devoid of CDI consistent with this mutant channel functioning without STIM1 (Fig. 7f–i,k,l). Thus, while STIM1 interaction with the Orai1 N terminus is not required for channel gating, such an interaction may have a regulatory role on channel function. We investigated whether perhaps STIM1 could rescue CDI activity in the Orai1-ANSGA mutant, however, even co-expressed with STIM1, store depletion did not reveal any CDI for Orai1-ANSGA (Fig. 7j,k). Since the binding of STIM1 to Orai1-ANSGA is substantially reduced, the direct action of STIM1 to restore CDI is difficult to assess using this mutant. However, the results indicate that CDI likely requires STIM1 binding to the C as well as the N termini of Orai1.

**Figure 8:**
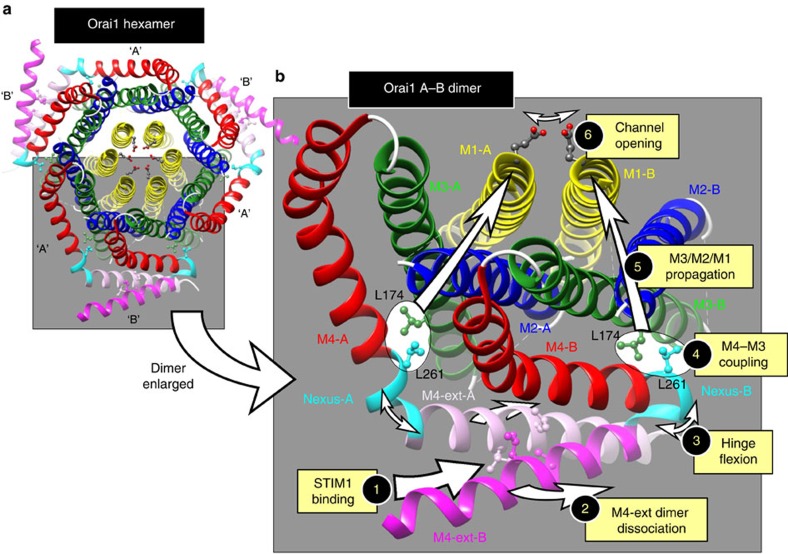
Postulated gating mechanism for Orai1 by STIM1. (**a**) The hexameric *Drosophila* Orai crystal structure^18^, comprising three pairs of Orai dimers. As described in Supplementary Fig. 1, dimers are formed from ‘A' and ‘B' subunits with identical sequence, differing only in the degree of bending of the nexus hinge. ‘A' subunits are only slightly bent and ‘B' subunits are bent almost 180° so that the two M4-ext sequences in the dimer pair lie antiparallel to each other, bound by hydrophobic interactions between the hOrai1 equivalent residues, L273 and L276. (**b**) Enlarged view of the Orai1 dimer showing the postulated gating mechanism activated by STIM1. STIM1 binding to the M4-ext pair (1) requires that the two M4-ext helices are in the conjoined antiparallel configuration, but they must be capable of undergoing dissociation for STIM1 to successfully bind to Orai1 (ref. 20). Likely, STIM1 binding induces some degree of dissociation between the two M4-ext helices (2). Such dissociation is permitted by the ability of the nexus ‘hinge' (263–265, SHK) to undergo flexion, and recent evidence reveals flexion of the hinge is required for STIM1-induced gating of the Orai1 channel^13^,^20^,^37^. Flexion of the hinge (3) likely induces conformational change in the adjoining ‘hinge-plate' residues (261–262; LV) of the M4 helix. The M3 helix L261 residue undergoes close hydrophobic interaction with the M3 helix L174 residue (4), hence displacement of the M4 residue induces a conformational change in M3. This conformational change is propagated through the tightly packed M3, M2 and M1 helices (5) to induce conformational alteration of the external entrance to the pore (6), as recently shown^12^.

The ANSGA construct provides a model system for determining the structural configuration of the Orai1 open channel. From its function we can postulate the mechanism of channel gating by STIM1. The binding of STIM1 to gate the channel is restricted to the C-terminal M4-ext, causing a discrete rearrangement in the adjacent nexus. The self-interacting M4-ext dimer between two adjacent Orai1 residues in the hexamer is required for STIM1 binding^20^. We propose a model (Fig. 8) in which the M4-ext dimer is ‘teased' apart by STIM1, resulting in flexion of each Orai1 monomer SHK hinge, strain on the LV-hinge plate, displacement of the closely associated M3-L174 residue and a discrete conformational change propagated through the M3/M2/M1 cluster, resulting in rearrangement of the extracellular pore mouth to open the channel. Thus, we hypothesize that STIM1 does not directly control the N-terminal Orai1 pore, instead the STIM1-bound Orai1 C terminus remotely controls outer pore gating through a nexus-mediated internal allosteric switch.

This model postulates a much simpler Orai1 channel gating mechanism than has previously been suggested^7^,^9^,^10^,^11^,^12^,^13^,^14^,^15^,^16^,^17^,^26^. Likely, the Orai1 channel can switch between two stable configurations, and the simple attachment of STIM1 to the C-terminal M4-ext is sufficient to induce this configurational change to the open state. It has been thought that the two Orai-activating sites within a STIM1 dimer bind across the two adjacent conjoined Orai1 monomers comprising the Orai1 dimer^2^,^30^. Interestingly, we recently revealed that although the active site of the STIM1 molecule must be in a dimeric configuration, it needs only to undergo a monomeric interaction with Orai1 to bind to and activate channel opening^19^. From this information and based on the current findings, it seems that Orai1 channel activation requires only monomeric interactions between STIM1 and the Orai1 C terminus, even though both STIM1 and Orai1 must exist as dimers to be functional.

## Methods

### DNA constructs

The CFP-Orai1 construct was made using human Orai1 obtained by PCR from the pDest-Orai1 template (from Dr James W. Putney, NIEHS-NIH)^31^ and subcloned into pECFP-C1 (Clontech) using XhoI/KpnI restriction sites. The Orai1-mRFP sequence was obtained by PCR from the Orai1-mRFP-FKBP12 template (from M.K. Korzeniowski, Cornell University and T. Balla, NICHD) and subcloned into pECFP-C1 using NheI/KpnI restriction sites. The Orai1-mRFP N-terminal deletion mutants (Orai1-ΔN74-mRFP and Orai1-ΔN77-mRFP) were obtained by PCR from Orai1-mRFP, and N-terminal NheI and C-terminal KpnI restriction sites for cloning into the pEYFP-C1 vector were added. The Orai1-mRFP C-terminal deletion mutant (Orai1-ΔC266-mRFP) was amplified via PCR from Orai1-mRFP, and N-terminal NheI and a C-terminal EcoRI restriction sites for cloning into the original vector were added. The CFP-Orai1-ΔN85 (amino acids 1–85 deleted) was made by Mutagenex, NJ using pDest-Orai1 as a template. All Orai1 mutations were generated using the QuikChange Lightning Site-Directed Mutagenesis Kit (Agilent Catalogue No. 210518). Primer information is given below in the primer list. All constructs were confirmed by sequencing before transfection.

### Cell culture and transfection

HEK293 cells (American Type Culture Collection CRL-1573, referred to here as ‘HEK' cells) were cultured in Dulbecco's modified Eagle's medium (Mediatech) supplemented with 10% fetal bovine serum, penicillin and streptomycin (Gemini Bioproducts, CA) at 37 °C with 5% CO_2_. Stable HEK cells expressing STIM1-YFP (HEK-STIM1-YFP) were derived^32^ and cultured in the same medium as above supplemented with purymicin (2 μg ml^−1^). Hela cells stably expressing GFP-NFAT1 (Hela-GFP-NFAT1 cells) were derived^33^ and cultured in the same medium as above supplemented with G418 (100 μg ml^−1^). All transfections were undertaken by electroporation at 180 V, 25 ms in 4 mm cuvettes (Molecular Bio-Products) using the Bio-Rad Gene Pulser Xcell system in OPTI-MEM medium. For cell lines transfected with constitutively active Orai1 channel mutants (ANSGA and other nexus mutants, V102C and P245L), following transfection cells were cultured in growth medium supplemented with 600 μM EGTA to reduce extracellular Ca^2+^. All experiments were performed 18–24 h after transfection.

### Cytosolic Ca^2+^ measurements

Cytosolic Ca^2+^ levels were measured by ratiometric imaging using fura-2 between 18–24 h after transfection^19^. Loading of fura-2 and imaging were performed in Ca^2+^-free solution containing (in mM): 107 NaCl; 7.2 KCl; 1.2 MgCl_2_; 11.5 glucose; and 20 HEPES-NaOH, pH 7.2. In experiments, 1 mM CaCl_2_ was added as indicated. Loading of cells with 2 mM fura-2/AM was for 30 min at room temperature, followed by treatment with fura-2-free solution for a further 30 min. Fluorescence ratio imaging was measured utilizing the Leica DMI 6000B fluorescence microscope and Hamamatsu camera ORCA-Flash 4 controlled by Slidebook 6.0 software (Intelligent Imaging Innovations; Denver, CO)^34^. Consecutive excitation at 340 nm (*F*_340_) and 380 nm (*F*_380_) was applied every 2 s and emission fluorescence was collected at 505 nm. Intracellular Ca^2+^ levels are shown as *F*_340_/*F*_380_ ratios obtained from groups of >35 single cells on coverslips. All Ca^2+^ imaging experiments were performed at room temperature and representative traces of at least three independent repeats are shown as means±s.e.m. Note that experiments were performed in HEK cells without overexpressed STIM1 (denoted as ‘HEK cells') or in HEK cells stably expressing STIM1-YFP (denoted as ‘HEK-STIM1 cells').

### FRET measurements

FRET was examined using a modified protocol^19^. FRET signals occurring between STIM1-YFP stably expressed in cells and CFP-Orai1-WT or CFP-Orai1 mutants transiently expressed were measured using a Leica DMI 6000B inverted automated fluorescence microscope that was equipped with CFP (438Ex/483Em), YFP (500Ex/542Em) and FRET (438Ex/542Em) filter cubes. During experiments, images were captured at 20 s intervals to avoid photobleaching. Three sets of images (CFP, YFP and FRET) were collected for each time point (room temperature) using a × 40 oil objective (numerical aperture 1.35; Leica). Images were processed using the Slidebook 6.0 software (Intelligent Imaging Innovations), images captured at intervals of 20 s. In each case, exposure times were 1,000, 250 and 1,000 ms for the CFP, YFP and FRET channels, respectively; the decreased channel exposure time for YFP compensates for the greater YFP fluorescence intensity as compared with CFP. Calculation of three-channel corrected FRET used the formula


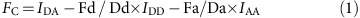


in which *I*_DD_, *I*_AA_ and *I*_DA_ are the intensities of background-subtracted CFP, YFP and FRET images, respectively, *F*_C_ is the corrected energy transfer, Fd/Dd is the measured bleed-through of CFP across the FRET filter (0.457) and Fa/Da is the measured bleed-through of YFP across the FRET filter (0.19). To analyse three-cube FRET images, we utilized the E-FRET method, which was described by Zal and Gascoigne^35^ using the formula:


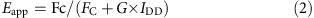


in which *G* is the instrument-specific constant^21^,^35^. We determined the value for *G* by measurement of the CFP fluorescence increase after YFP acceptor photobleaching in HEK cells that were transiently transfected with the pEYFP-ECFP construct^19^. We calculated the value of *G* as 1.9±0.1 (*n*=32 cells). For studies determining E-FRET between YFP and CFP constructs, cells with a narrow range of YFP/CFP ratios were selected to ensure comparability between measurements. In all our E-FRET summary data, a region close to the PM was selected, and E-FRET analysis was conducted on cells with similar YFP/CFP ratios. For E-FRET measurements following diamide addition, the diamide was washed out before collection of post-diamide FRET images to avoid interference from diamide fluorescence.

### Enhanced fluorescence image analysis

The inverted Leica DMI 6000B automated fluorescence microscope and Hamamatsu camera ORCA-Flash 4 controlled by Slidebook software (as above) were used to collect and analyse high-resolution fluorescence images. For the ER–PM co-localization studies of STIM1-YFP and CFP-Orai1 (WT or mutants) after store depletion by ionomycin, the stacks of 10–20 three-dimensional *Z*-axis image planes closed to the cell–glass interface were collected at 0.35 μm steps. For images of the PM distribution of Orai1 (CFP- or mRFP-tagged, WT or mutant), the no-neighbour deconvolution function of the Slidebook 6.0 software was used to analyse images and derive enhanced deconvolved images with minimized fluorescence contamination from out-of-focus planes. STIM1-YFP, CFP-Orai1, Orai1-mRFP and all other mutant images shown were typical of at least three independent experiments.

### Electrophysiological measurements

We performed patch-clamp recordings on HEK-STIM1 cells that transiently expressed CFP- or mRFP-tagged Orai1 mutants. Cells were cultured for one day before analyses. To maintain an ER store-replete state, the pipette solution comprised the following (in mM): 135 Cs-Aspartate; 10 HEPES; 4 MgCl_2_; 10 EGTA; and 3.6 CaCl_2_ (pH 7.2 with CsOH). The quantity of EGTA and CaCl_2_ were determined using WEBMAXCLITE (http://web.stanford.edu/~cpatton/webmaxc2) to maintain cytosolic Ca^2+^ at ∼90 nM throughout experiments. Passive ER store depletion was induced using a pipette solution containing the following (in mM): 135 Cs-Aspartate; 10 HEPES; 8 MgCl_2_ and 10 BAPTA (pH 7.2 with CsOH). The bath solution contained 20 mM Ca^2+^ with the following (in mM): 130 NaCl; 4.5 KCl; 5.0 HEPES; 10 dextrose; 10 TEA-Cl; and 20 CaCl_2_ (pH 7.4 with NaOH). For Ca^2+^-free conditions, the bath solution contained (in mM) 150 NaCl; 4.5 KCl; 5.0 HEPES; 10 dextrose; 10 TEA-Cl; and 3 mM MgCl_2_ (pH 7.4 with NaOH). The standard whole-cell configuration was used to determine currents using an EPC-10 amplifier (HEKA). The typical resistance of glass electrodes was 2–4 MΩ; the electrodes were pulled using a P-97 pipette puller (Sutter Instrument). Every 2 s during recordings, we delivered a 50 ms step to −100 mV from a holding potential of 0 mV, followed by a 50 ms ramp from −100 to 100 mV. Currents were sampled at 20 kHz and filtered at 3.0 kHz. We applied a +10 mV junction potential compensation to correct the liquid junction potential between the bath and pipette solutions. Leak-free currents were obtained by subtracting currents recorded before Ca^2+^ stores were emptied (for Orai1+STIM1) or in Ca^2+^-free solution (for Orai1-ANSGA). For CDI experiments, a series of 200 ms voltage steps to −60, −80, −100 and −120 mV from a holding potential of 30 mV with a 5 s interval was applied. The voltage steps were applied to ANSGA 3 min after break-in to ensure the replacement of cytosol with pipette solution. We observed a mixture of capacitance transients and CRAC currents over the first 2 ms of recorded traces. Therefore, we measured the current amplitude at 2 ms to minimize the impact of capacitance transients. CDI was quantified as the current measured at 185 ms divided by the current measured at 2 ms. For all experiments, data were acquired with the Patch Master software and analysed afterwards using FitMaster and Prism programmes. Experiments were performed either in HEK cells without overexpressed STIM1 (denoted as ‘HEK cells') or in HEK cells stably expressing STIM1-YFP (denoted as ‘HEK-STIM1 cells').

### Western blot and biotinylation analyses

Cell lysis was undertaken with pre-chilled lysis buffer containing 150 mM NaCl, 10 mM Tris-HCl (pH 7.4), 1% NP-40, with one tablet of complete protease inhibitors (Santa Cruz, sc-29131) per 25 ml. Lysis occurred on ice for 30 min, followed by centrifugation at 14,000 *g* for 10 min at 4 °C; supernatant protein determined using Bio-Rad DC protein assay kits. The protein extracts so obtained were resolved on 4–12% NuPAGE Bis-Tris precast gels (Life Technologies; 27 μg per lane of each), then transferred to Bio-Rad Immuno-Blot polyvinylidene difluoride membranes (162–0177, Bio Rad). Following transfer, polyvinylidene difluoride membranes were blocked using phosphate-buffered saline–Tween 20 (PBST; containing 1 × PBS (46–013-CM, Mediatech) and 0.1% Tween 20) together with 5% non-fat dried milk (M0841, LabScientific) at room temperature for 1 h. Thereafter, membranes were incubated overnight at 4 °C with mouse GFP primary antibody (SC9996, Santa Cruz) at a final concentration of 1 μg ml^-1^. Membranes were then washed for 7 min (three times) with PBST followed by incubation for 30 min at room temperature, with secondary antibody (Anti-Mouse immunoglobulin G (GE healthcare, NA931V) diluted 1:4,000). Following this, membranes were washed for 5 min (three times) with PBST. Peroxidase activity was determined with SuperSignal West Pico Chemiluminescent Substrate (Thermo Scientific) according to the manufacturer's protocol; the resulting fluorescence was imaged using a FluorChem M from ProteinSimple. The Pierce Cell Surface Protein Isolation kit (#89881) was used to isolate cell surface proteins via biotinylation. HEK cells were transfected with CFP-Orai1-WT or mutants 24 h before lysis, and cells were washed twice with ice-cold PBS and incubated with 0.25 mg ml^-1^ Sulfo-NHS-SS-Biotin for 30 min at 4 °C. Thereafter, quenching solution (#1859386) was added to stop the reaction. Cells are collected and rinsed with Tris-buffered saline. Cells were lysed with Lysis Buffer (#1859387) on ice for 30 min and centrifuged at 10,000 *g* for 10 min at 4 °C. The supernatant containing biotinylated proteins was incubated with Immobilized NeutrAvidin Gel (#1859388) in the columns at room temperature for 1 h on a rocking platform. After centrifuging at 1,000 *g* for 2 min, flow-through from the columns was loaded for GAPDH determination. Columns were wash three times with Wash Buffer (#1859389), and the biotinylated protein was eluted with SDS–PAGE Sample Buffer with 50 mM dithiothreitol and resolved on 4–12% NuPAGE Bis-Trisprecast gels (Life Technologies) as described above.

### Structural modelling

In images shown, the Rotamer Function of the Chimera software^36^ was used to predict orientation of amino acids lysine 163, threonine 246 and isoleucine 316 in *Drosophila* Orai1, with the human Orai1 equivalents (Arg 91, Leu 174 and Leu 273), respectively. The position of the side chains of substituted amino acids were modelled using the Dunbrack library using Chimera software. Scores with highest probability were used. Lysine 308 in *Drosophila* Orai1 (equivalent to Lys 265 in human Orai1) is not resolved in the study of Hou *et al*.^18^, and the same rotamer prediction as above was used to model the position of the side chain of Lys 265.

### Data availability

The data that support the findings of this study are available from the corresponding author on reasonable request.

## Additional information

**How to cite this article:** Zhou, Y. *et al*. The STIM1 binding site nexus remotely controls orai1 channel gating. *Nat. Commun.* **7,** 13725 doi: 10.1038/ncomms13725 (2016).

**Publisher's note:** Springer Nature remains neutral with regard to jurisdictional claims in published maps and institutional affiliations.

## Supplementary Material

Supplementary Information Supplementary Figures 1-8, Supplementary Tables 1-4, Supplementary References.
