# Supplementary material for: The STIM1-binding site nexus remotely controls Orai1 channel gating
Source: Nat Commun. 2016 Dec 8;7:13725. doi: 10.1038/ncomms13725 (PMC5155162; doi:10.1038/ncomms13725)
Supplement: Supplementary Information — Supplementary Figures 1-8, Supplementary Tables 1-4, Supplementary References. [file ncomms13725-s1.pdf]

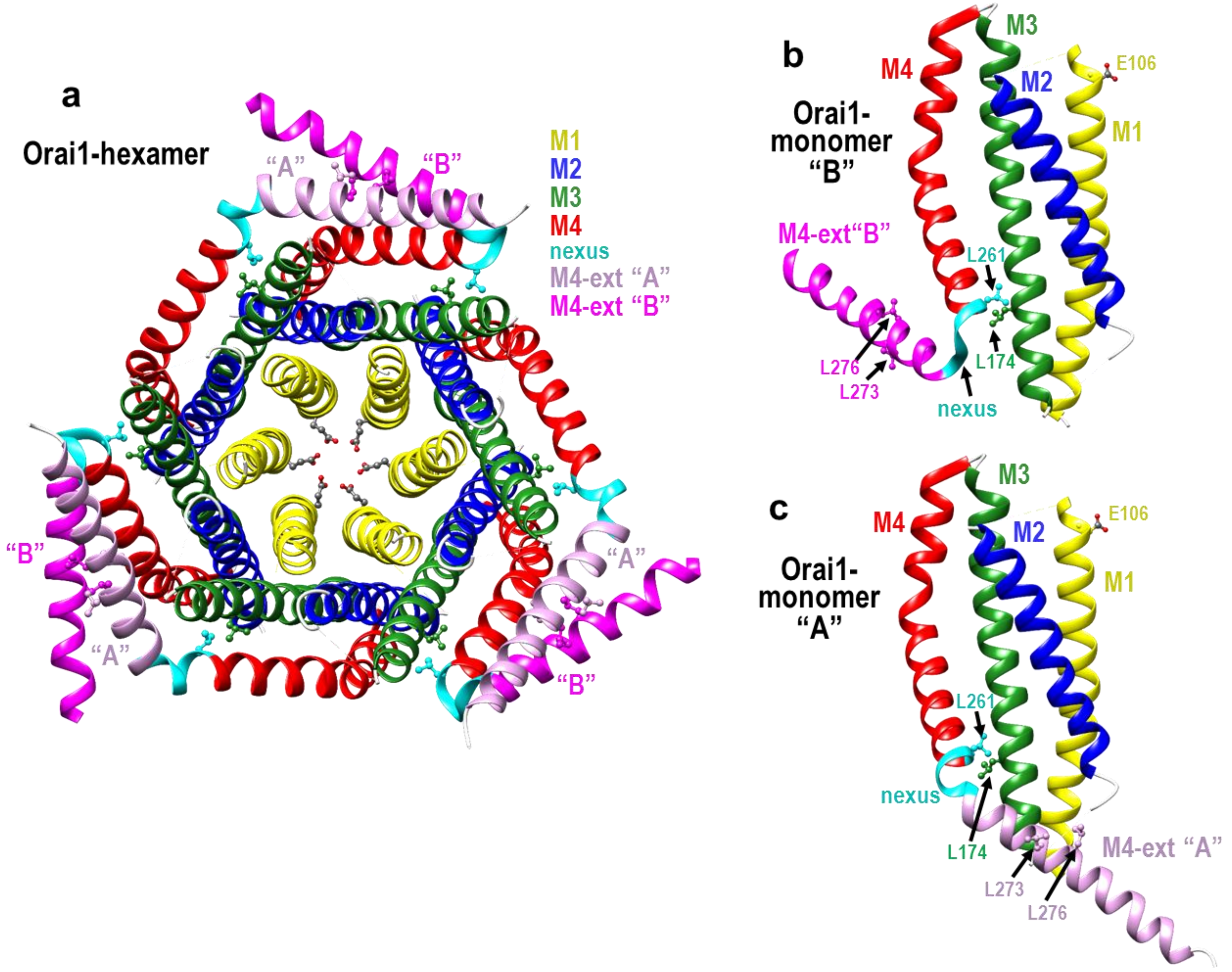

Supplementary Figure 1 │ Structure of the Orai channel. (**a**) The hexameric *Drosophila* Orai channel structure derived from crystallography[1] comprises six Orai subunits, each with identical amino acid sequences including four transmembrane (TM) helices (M1 – M4). The TM helices are almost identical in human Orai1. Viewed from the cytoplasmic face, the six N-terminal M1 TM helices (yellow) line the central channel pore. The selectivity filter at the outer pore mouth is formed by the ring of glutamate residues shown (E106 in hOrai1)[2]. The M1 pore is surrounded by the tightly-packed M2 (blue) and M3 (green) TM helices. The C-terminal M4 TM helix (red) lies at the periphery and more distant from the other helices. The M4 helix is linked by a five amino acid "nexus" sequence (cyan) to the cytoplasmic M4-extension (M4-ext) helices (purple or pink), which form strong cytoplasmic binding sites for STIM1. The six Orai1 subunits are arranged as three dimers, each dimer pair comprising one "A" and one "B" Orai1

subunit isoform. The structure of the “B” and “A” dOrai monomer isoforms are shown in their side configuration in (**b**) and (**c**). Although the “A” and “B” isoforms have identical sequences, they differ in the degree of bending at the nexus. In the “A” subunits, the nexus is only slightly bent, whereas in “B” subunits the nexus is bent almost 180°. This allows the two adjacent M4-ext helices within each dimer pair to lie in a closely aligned antiparallel configuration bound together by hydrophobic interactions between the human Orai1 equivalent residues, L273 and L276 as shown in (a). The antiparallel interactions between the two M4-ext sequences appear to be crucial for allowing STIM1 binding to Orai1[3]. The nexus region (Orai1 261-265, LVSHK) plays a critical role in Orai1 gating. The SHK residues (263-265) provide a conserved, flexible hinge between the M4 and M4-ext helices[1], and flexion of this hinge is shown to be crucial for STIM1-induced gating of Orai1[3,4]. The LV residues (261-262) are conserved in all Orai subtypes and species. The L261 residue lies in close apposition to L174 in M3 and forms a crucial hydrophobic point of contact between the STIM1 binding M4-ext and the tightly-packed M3, M2, and M1 TM helices. Important to note, the configuration of the M4-L261 and its interaction with the M3-L174 are almost identical in both the “A” and “B” isoforms of Orai1 (b, c). Subsequent residues C-terminal of L261 become progressively different in configuration between the “A” and “B” conformers. Thus, the nexus serves as the crucial transition between the 6-fold symmetry of the entire M1-M4 helical structure, and the 3-fold symmetry of the conjoined M4-ext helices.

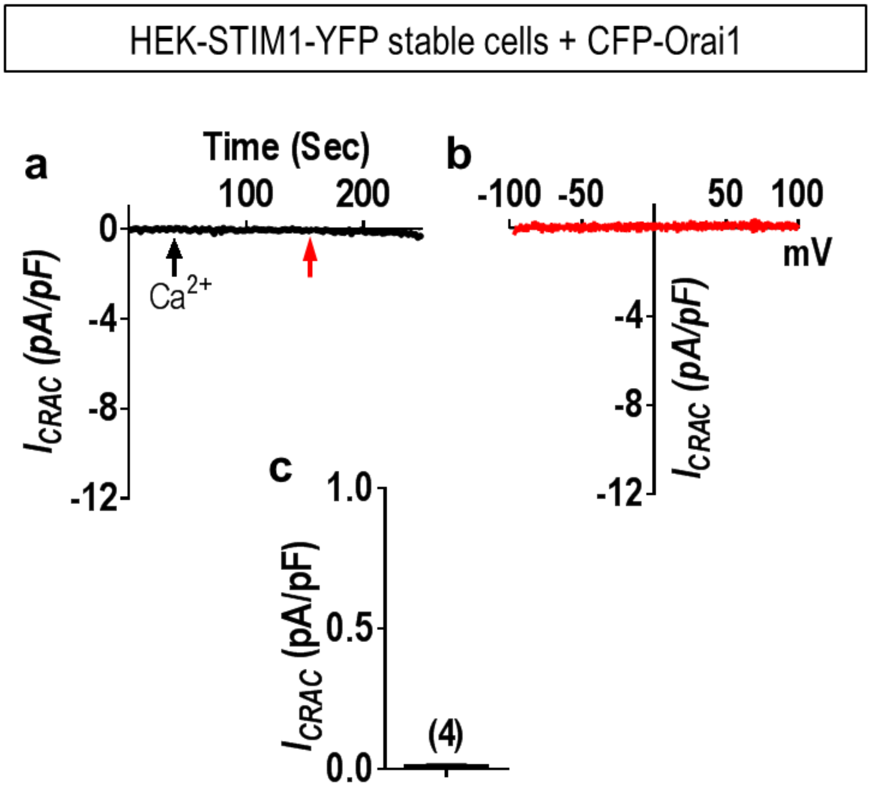

Supplementary Figure 2 │ Control experiments showing, under store-replete conditions, HEK-STIM1-YFP cells expressing CFP-Orai1 do not develop I_{CRAC}. (**a**) The CFP-Orai1-WT channel is not active in HEK-STIM1 cells when cytosolic Ca^{2+} is buffered to 90 nM with EGTA (see Methods), even 200 seconds after break-in. (**b**) I-V plot for the same cell in (a). (**c**) Summary of the peak current amplitudes measured at 150 sec after break-in. Values are mean ± SEM, n=4.

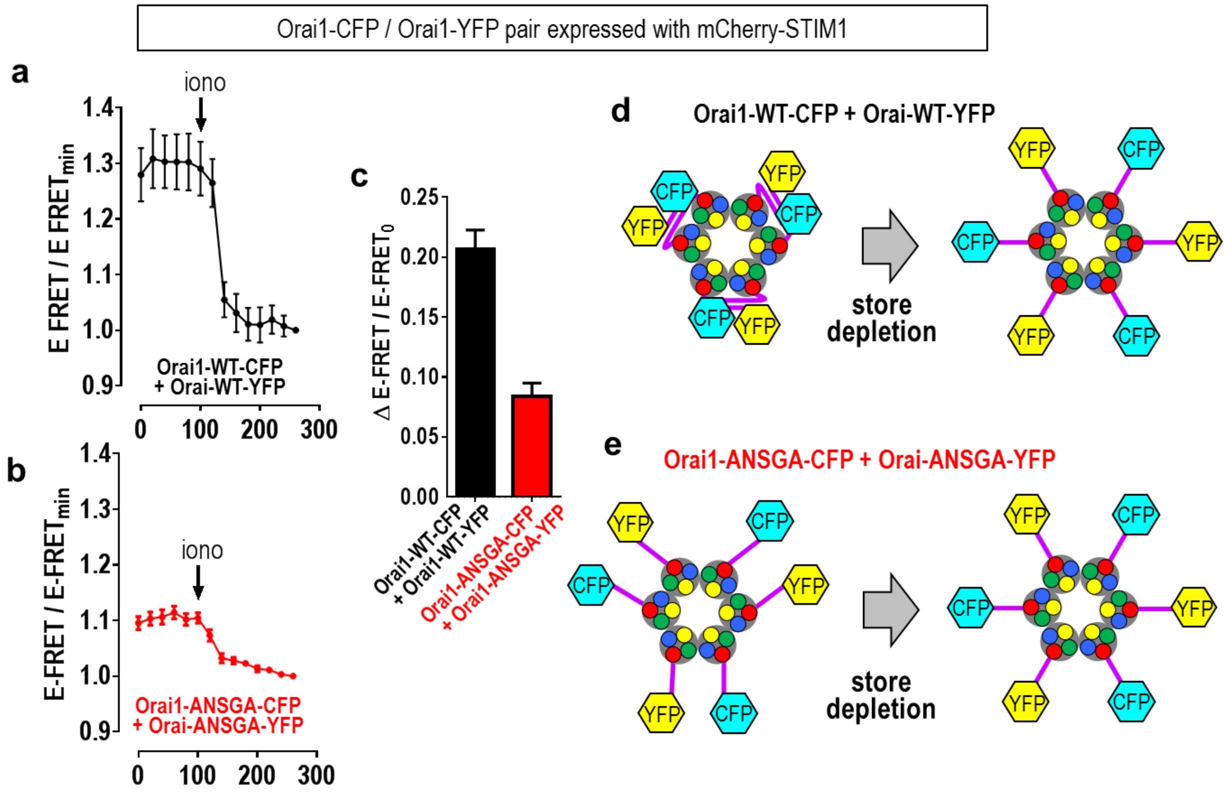

Supplementary Figure 3 │ Orai1-Orai1 FRET changes after store-depletion are considerably reduced in the Orai1-ANSGA mutation compared to Orai-WT. (**a**) E-FRET change was measured in HEK cells expressing a mixture of C-terminally-labeled Orai1-WT-CFP and Orai1-WT-YFP together with mCherry-STIM1, and E-FRET observed in response to store-depletion with 2.5 μM ionomycin. E-FRET values were normalized to final minimum levels after ionomycin. (**b**) E-FRET changes in HEK cells expressing a mixture of Orai1-ANSGA-CFP and Orai1-ANSGA-YFP together and mCherry-STIM1 in response to 2.5μM ionomycin, as determined in (a). (**c**) Summary of YFP-CFP E-FRET changes for the Orai1-WT and Orai1-ANSGA data shown in (a) and (b); WT, n=38; ANSGA, n=37. Values are means ± SEM. (**d**) Hypothetical model for WT Orai1-Orai1 FRET decrease upon store depletion. The Orai1-WT channel is shown in its resting state with M4-ext helices within Orai1 dimer pairs in close proximity. Following store-depletion, the rearrangement of the M4-ext helices due to STIM1 binding leads to a decrease in Orai1-Orai1-FRET. (**e**) As in **d**, but using ANSGA Orai1-Orai1 FRET, the M4-ext helices in the resting state (without store-depletion) are in an altered, displaced configuration, perhaps reflecting the activated state of the channel. Upon store-depletion, there is little rearrangement of the M4-ext helices due to STIM1 binding.

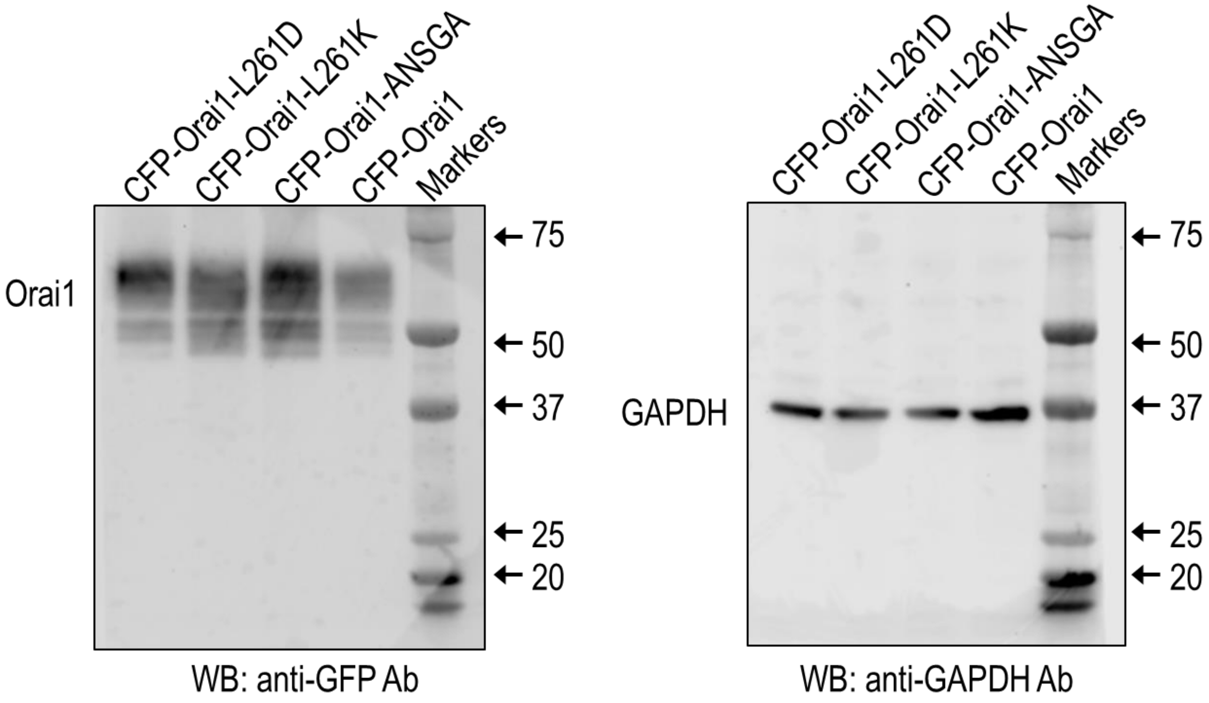

Supplementary Figure 4 │ Plasma membrane expression of CFP-Orai1 mutants in HEK cells. CFP-Orai1, CFP-Orai1-L261K, CFP-Orai1-L261D or CFP-Orai1-ANSGA were expressed in HEK cells and plasma membrane protein isolated by biotinylation and determined by western blot as described in Methods.

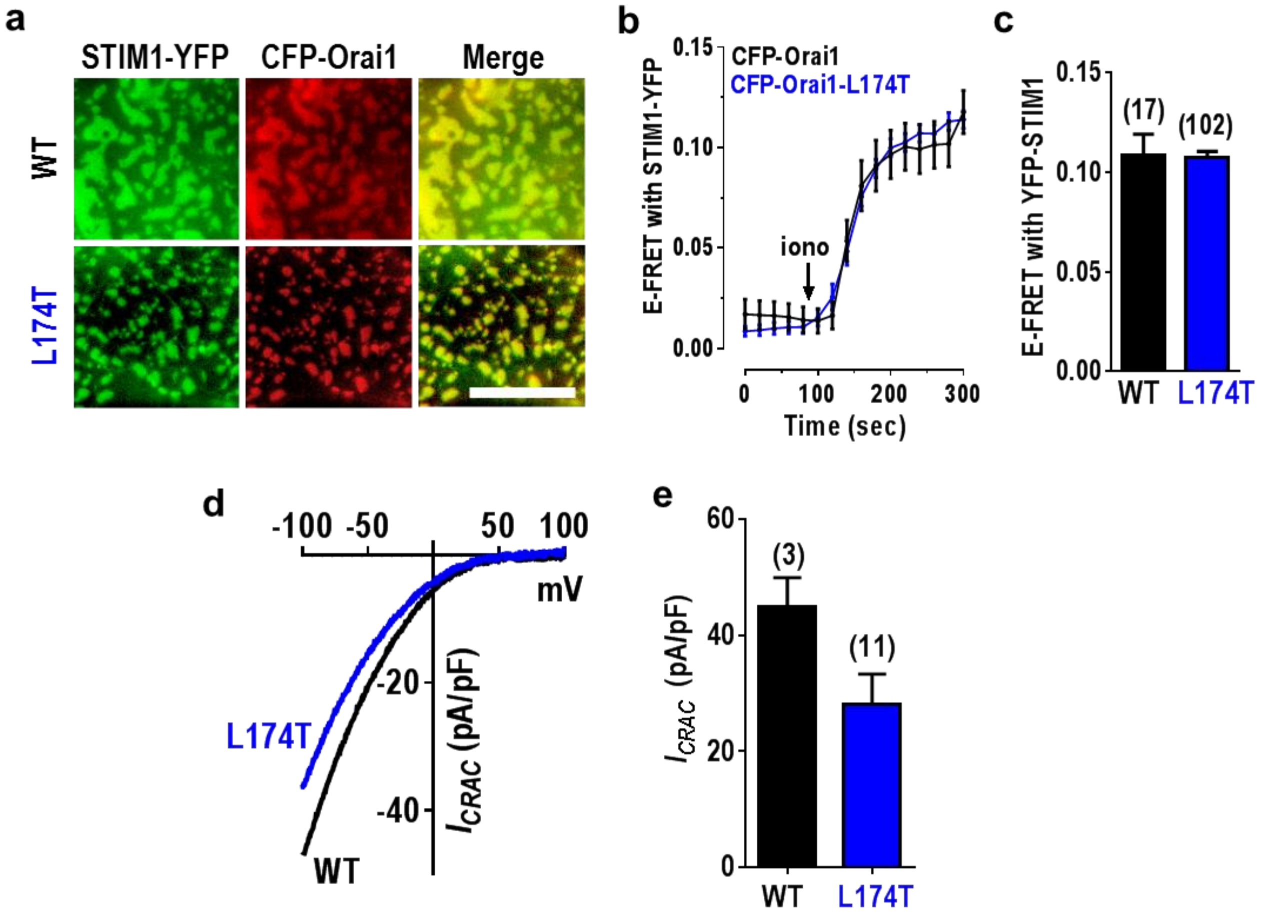

Supplementary Figure 5 │ The L174T mutation in Orai1 does not inhibit its association with STIM1 and only modestly reduces channel activation by STIM1. (**a**) High-resolution imaging of the ER-PM interface in HEK-STIM1-YFP cells transiently expressing CFP-Orai1-WT or CFP-Orai1-L174T after 2.5μM ionomycin treatment. Scale bar = 5 μm. (**b**) E-FRET between STIM1-YFP and CFP-Orai1 or CFP-Orai1-L174T expressed in HEK-STIM1-YFP cells. (**c**) Summary of E-FRET peak values after store-depletion as shown in (b). (**d**) I–V relationship for stable HEK-STIM1-YFP cells expressing CFP-Orai1 (black) or CFP-Orai1-L174T (blue). (**e**) Summary of peak current amplitudes measured at -100 mV shown in (d). Values are means ± SEM.

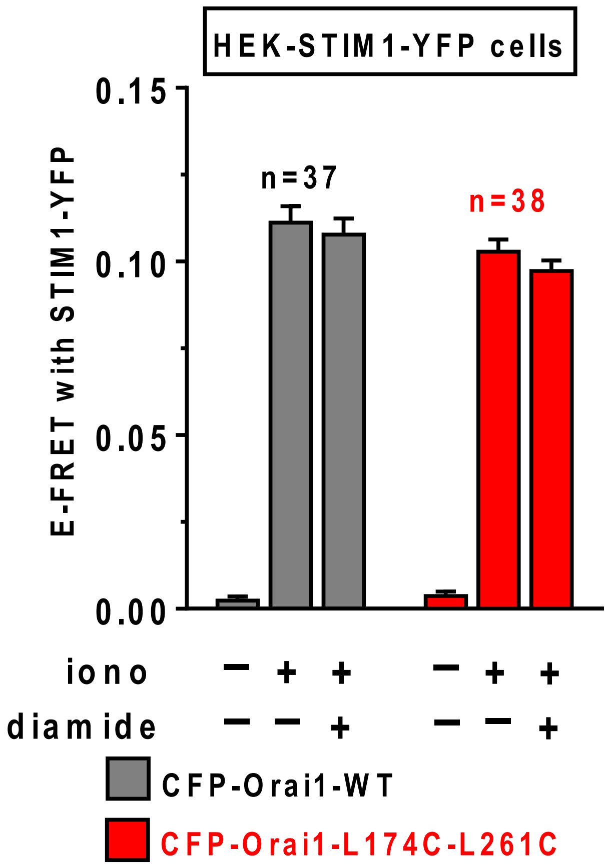

Supplementary Figure 6 │ Diamide addition does not alter the binding of Orai1-WT or the CFP-Orai1-L174C-L261C with STIM1-YFP, as measured by FRET. CFP-Orai1-WT (gray) or CFP-Orai1-L174C-L261C (red) were expressed in HEK-STIM1-YFP cells. 24 hr after transfection, E-FRET was measured either before or after addition of 2.5 μM ionomycin (iono) for 5 min to empty stores, followed by addition of 500 μM diamide. After 2 min with diamide, final FRET images were obtained immediately after washing with diamide-free Ringer's solution to eliminate diamide fluorescence. Values are means ± SEM.

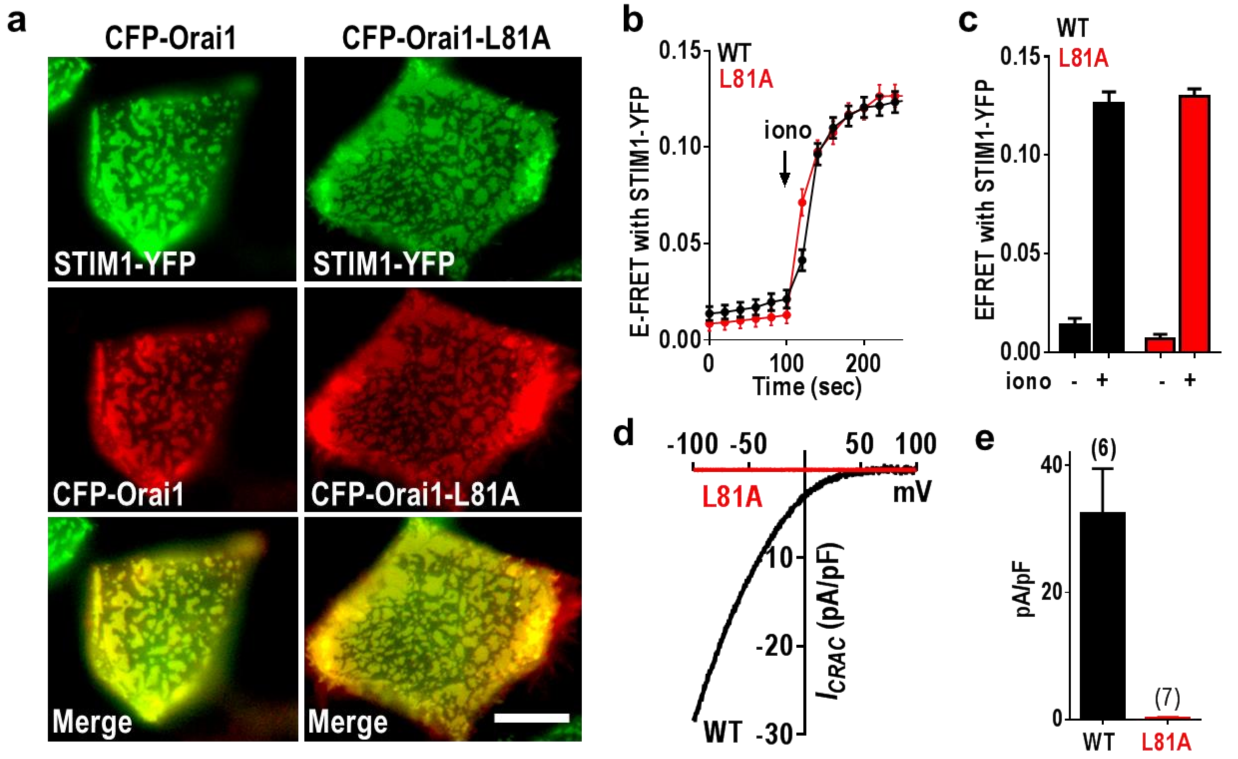

Supplementary Figure 7 | The Orai1 N-terminal mutation, L81A, completely blocks STIM1-induced channel activation without altering coupling with STIM1. (**a**) High-resolution imaging of the ER-PM interface in HEK-STIM1-YFP cells transiently expressing WT or L81A after 2.5µM ionomycin treatment. Scale bar = 5 µm. (**b**) E-FRET between STIM1-YFP and CFP-Orai1 or CFP-Orai1-L81 expressed in HEK-STIM1-YFP cells as in (a). (**c**) Summary of E-FRET peak values after store-depletion for the Orai1 mutants shown for (b). (**d**) I–V relationship for stable HEK-STIM1-YFP cells expressing CFP-Orai1 (black) or CFP-Orai1-L81A (red). (**e**) Summary of the peak current amplitudes measured at -100 mV shown in (d). Values are means ± SEM.

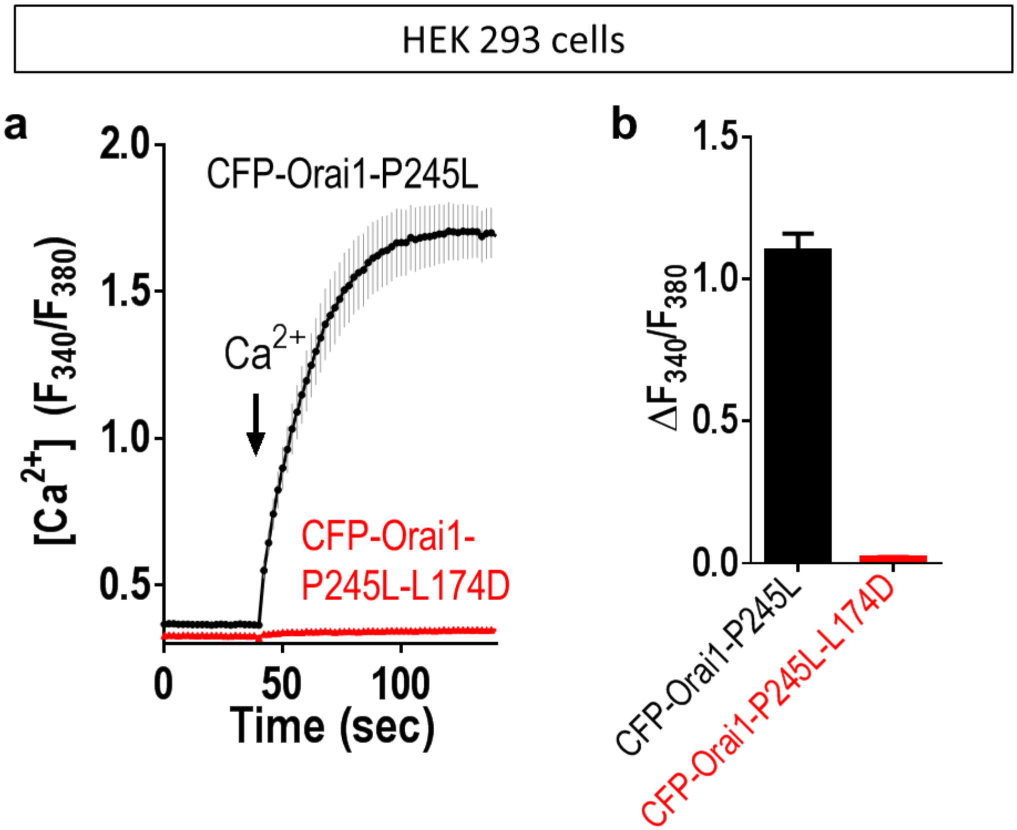

Supplementary Figure 8 │ The Orai1 M3-helix L174D mutation completely abolishes the constitutive Ca^{2+} entry mediated by the Orai1 M4-helix mutant, CFP-Orai1-P245L. (**a**) Fura-2 Ca^{2+} responses in store-replete HEK cells expressing CFP-Orai1-P245L (black) or CFP-Orai1-P245L-L174D (red). (**b**) Summary of the constitutive Ca^{2+} entry in cells shown in (a)**.** Values are mean ± SEM.

Supplementary Table 1. Primer sequences for used for full-length and truncation constructs of Orai1

CFP-Orai1	forward	CCGCTCGAGCTACCATGCATCCGGAGCCCGC;
	reverse	CGGGGTACCCTAGGCATAGTGGCTGCCGGG
Orai1-mRFP	forward	CTAGCTAGCGGGATATCGCTCCATGCATC;
	reverse	GCTCTAGACTA AGATCTGAGTCCGGAGGCGCC
Orai1-ΔN74-mRFP	forward	CTAGCTAGCATGTCCTGGCGCAAGCTCTAC
	reverse	CGGGGTACCCTAAGATCTGAGTCCGGAG GCGCC
Orai1-ΔN77-mRFP	forward	CTAGCTAGCATGAAGCTCTACTTGAGCCGCGCC
	reverse	CGGGGTACCCTAAGATCTGAG TCCGGAGGCGCC.

Supplementary Table 2. Primer sequences for used for L261-V262-S263-H264-K265 multiple mutations (only forward primers shown)

AVSHK	CCACTTCTACCGCTCAGCGGTTAGCCATAAGACTGACC
LNSHK	CCACTTCTACCGCTCACTGAATAGCCATAAGACTGACCG
ANSHK	CGTCCACTTCTACCGCTCAGCGAATAGCCATAAGACTGACCG
ANSGK	CCACTTCTACCGCTCAGCGAATAGCGGTAAGACTGACCGACAGTTCC
ANSHA	CCGCTCAGCGAATAGCCATGCGACTGACCG
ANSGA	GCCGTCCACTTCTACCGCTCAGCGAATAGCGGTGCGACTGACCGACAGTTCCA
AKSGA	CCGTCCACTTCTACCGCTCAGCGAAGAGCGGTGCGACTGACCGACAGTTCCAGG
AKSHK	CCACTTCTACCGCTCAGCGAAGAGCCATAAGACTGACCGACAGTTCC
ADSHK	CCACTTCTACCGCTCAGCGGATAGCCATAAGACTGACC
AVSHA	CCACTTCTACCGCTCAGCGGTTAGCCATGCGACTGACCG
LVSGA	CCGCTCACTGGTTAGCGGTGCGACTGACCGACAGTTCC
GDSHK	CCACTTCTACCGCTCAGGGGATAGCCATAAGACTGACC
GNSHK	CGTCCACTTCTACCGCTCAGGGAATAGCCATAAGACTGACCG
AAAAA	CCGTCCACTTCTACCGCTCAGCGGCTGCCGCTGCGACTGACCGACAGTTCC

Supplementary Table 3. Primer sequences for used for L81-S82-R83-A84-K85 multiple mutations (only forward primers shown)

AARAE	CCTGGCGCAAGCTCTACGCGGCACGAGCCGAGCTTAAAGCCTCC

Supplementary Table 4. Primer sequences for used for single amino acid mutations (only forward primers shown)

L261K	CCACTTCTACCGCTCAAAGGTTAGCCATAAGACTGACC
L261D	CCACTTCTACCGCTCAGACGTTAGCCATAAGACTGACC
L174K	GCCACATCGAGAAGGCCTGGGCCTTCTCC
L174D	CCGCCACATCGAGGACGCCTGGGCCTTCTCC
A175K	CGCCACATCGAGCTGAAGTGGGCCTTCTCCACC
L273D	CGACAGTTCCAGGAGGACAACGAGCTGGCG
L174C	CCGCCACATCGAGTGCGCCTGGGCCTTCTCC
L261C	CCACTTCTACCGCTCATGCGTTAGCCATAAGACTGACCG
V102C	CGGCTTCGCCATGTGCGCAATGGTGGAGGTGC
E106A	GGTGGCAATGGTGGCGGTGCAGCTGG
R91W	GCTTAAAGCCTCCAGCTGGACCTCGGC
R83A/K87A	GCTCTACTTGAGCGCAGCCAAGCTTGCAGCCTCCAGCCFGG
K85E	GCCGCGCCGAGCTTAAAGCCTCCAGC

Supplementary References

1. Hou, X., Pedi, L., Diver, M. M. & Long, S. B. Crystal structure of the calcium release-activated calcium channel Orai. *Science* **338**, 1308-1313 (2012).

2. Prakriya, M. & Lewis, R. S. Store-Operated Calcium Channels. *Physiol Rev* **95**, 1383-1436 (2015).

3. Tirado-Lee, L., Yamashita, M. & Prakriya, M. Conformational Changes in the Orai1 C-Terminus Evoked by STIM1 Binding. *PLoS One* **10**, e0128622 (2015).

4. Palty, R., Stanley, C. & Isacoff, E. Y. Critical role for Orai1 C-terminal domain and TM4 in CRAC channel gating. *Cell Res* **25**, 963-980 (2015).
